# Therapeutic potential of haptoglobin in a murine model of sickle cell anemia

**DOI:** 10.1371/journal.pone.0357180

**Published:** 2026-09-18

**Authors:** Bianca Cristina dos Santos, Magnun Nueldo Nunes dos Santos, Gisele Audrei Pedroso, Beatriz Benedetti de Oliveira, Dulcinéia Martins de Albuquerque, Nathalia Rocha de Oliveira, Daniela Cagnoto Noronha, Fernando Ferreira Costa, Marcus Alexandre Finzi Corat

**Affiliations:** 1 Laboratory for the Development of Biological Models, Multidisciplinary Center for Biological Research, University of Campinas (UNICAMP), Campinas, SP, Brazil; 2 Department of Pathology, Hemoglobinopathies Laboratory, School of Medical Sciences, University of Campinas (UNICAMP), Campinas, SP, Brazil; 3 Department of Pathology, Hematology Laboratory, Clinical Pathology Laboratory (LPC), University Hospital, School of Medical Sciences, University of Campinas (UNICAMP), Campinas, SP, Brazil; 4 Center for Hematology and Hemotherapy, National Institute of Science and Technology in Blood (INCTS), University of Campinas (UNICAMP), Campinas, SP, Brazil; Sao Francisco University: Universidade Sao Francisco, BRAZIL

## Abstract

Chronic hemolytic anemia found in patients with sickle cell disease promotes an excess of free extracellular and intravascular hemoglobin, triggering a series of deleterious effects such as endothelial dysfunction, oxidative stress, and alterations in vascular tone, which lead to clinical complications. In this work, we evaluated the induced expression of haptoglobin in hepatocytes in vivo, the organism’s first line of defense to control the presence of free Hb in circulation, which becomes saturated in conditions of chronic hemolysis, such as sickle cell disease. Through gene therapy applied in a murine model of sickle cell anemia, we aimed to investigate whether this approach could interfere with disease progression and promote an improvement in the animals’ pathophysiological condition. The application of this treatment in the sickle cell anemia animal model resulted in significant therapeutic effects, including improvement of hematological parameters, reduction of the compensatory erythropoietic response, and attenuation of clinical signs such as splenomegaly. These results indicate that haptoglobin may act indirectly in the prevention of hemolysis, suggesting a new therapeutic approach.

## Introduction

Sickle cell disease (SCD) is a genetic hemoglobinopathy that causes a morphological and functional variation in red blood cells. Patients present a point mutation responsible for the substitution of a base in codon 6 of the β-globin gene. In this case, an adenine is replaced by a thymine in the sequence, resulting in the substitution of the amino acid glutamate by valine, leading to the formation of hemoglobin S (HbS). Under hypoxic conditions, HbS polymerizes, generating a cellular deformation that makes the red blood cells elongated and sickle-shaped. This hinders their passage through the capillaries, leading to vascular obstructions, tissue ischemia, and frequent pain crises [1–4].

These sickled cells also have a reduced lifespan compared to normal erythrocytes, and this premature destruction causes a chronic hemolytic anemia condition. The accelerated destruction of erythrocytes is described as hemolysis and is a central component of the disease’s pathophysiology. During hemolysis, there is a massive release of free hemoglobin into the bloodstream. This extracellular hemoglobin, when not properly captured by natural clearance mechanisms, can trigger a series of deleterious effects, such as endothelial dysfunction, oxidative stress, and changes in vascular tone, further worsening the clinical complications of SCD [4].

The first line of defense to control the presence of free Hb in the circulation is through the action of haptoglobin (Hp). Hp is a plasma protein synthesized by the liver and was located on the long arm of chromosome 16 in 1969 [5]. It has two main alleles (Hp1 and Hp2), which by dimerization determine three distinct phenotypes (Hp1−1; Hp2−1; and Hp2−2) [6].

Besides its origin and genetic variations, the main function of haptoglobin is to bind specifically and with high affinity to hemoglobin released during intravascular hemolysis, forming a haptoglobin-hemoglobin complex (Hp -Hb) [7,8] By binding to free Hb, this interaction prevents it from circulating to organs like the kidneys, where its activity may lead to cellular injury. This is because the formed complex is rapidly recognized by macrophages in the liver and spleen, being removed from circulation through the rapid binding of the Hb-Hp complex to the CD163 receptor found on macrophages, which internalize the free hemoglobin and initiate proteolytic degradation of hemoglobin and heme catabolism, preventing the oxidation of Fe2+ [7,9]. This mechanism is essential to prevent the toxicity associated with free hemoglobin.

In addition to the hemolytic protection systems related to haptoglobin, there are also more downstream protective mechanisms in the iron oxidation process, such as the hemopexin/ heme oxygenase/biliverdin reductase pathway, which primarily acts on heme degradation, and transferrin, which acts directly on iron already oxidized to Fe3 + , preventing its further oxidation to the more toxic Fe4 + state [10,11]. However, under pathological conditions with sustained intense and chronic hemolysis, leading to continuously increasing levels of free hemoglobin, as occurs in SCD, these clearance systems become saturated, resulting in the progressive accumulation of free hemoglobin in the circulation. This leads to the oxidation of Fe2+ to Fe4 + , worsening the vascular and inflammatory complications characteristic of the disease [12,13].

With the aim of enhancing the first line of hemolytic defense, this study chose to promote the overexpression of haptoglobin 1 in order to evaluate the organism’s response to this treatment during the development of sickle cell disease. The choice of haptoglobin phenotype Hp1 was based on literature evidence demonstrating the greater efficiency of this phenotype in neutralizing the deleterious effects of free hemoglobin. Studies indicate that Hp1−1 exhibits higher antioxidant capacity compared to Hp2−2, promoting the formation of more stable Hb-Hp complexes with more efficient clearance via endocytosis. In contrast, complexes formed with Hp2−2 are associated with increased generation of reactive oxygen species, thereby enhancing the potential for oxidative damage to lipids, proteins, and DNA. Based on this evidence, the hypothesis is proposed that increasing circulating levels of haptoglobin may favor the formation of the Hb-Hp complex and, consequently, promote a more efficient removal of free hemoglobin. This approach could minimize the harmful effects of chronic hemolysis and thus represent a promising alternative to reduce the severity of the clinical manifestations of the disease. Therefore, considering the context of chronic hemolysis observed in sickle cell anemia, Hp1 was considered more appropriate as a therapeutic strategy due to its greater efficiency in reducing oxidative stress and its potential tissue-protective effects.

## Materials and methods

### Bone marrow transplantation to generate SCD mouse models

In this study, a mouse model with SCD (SCM) was adopted, generated by transplanting bone marrow from Berkeley SCM model, which is homozygous sickle cell mice STOCK (Hbatm1Paz Hbbtm1Tow Tg(HBA-HBBs)41Paz/J), as described by Reddy [14,15]. This approach allowed for the generation of mice with hematological characteristics compatible with SCD, through the replacement of the recipients’ hematopoietic stem cells with cells from donors carrying the sickle cell genotype. The bone marrow donor mice used in this study belong to the Berkeley model, a widely used humanized model of sickle cell disease that predominantly expresses human hemoglobin S. Additionally, the irradiated recipient animals belong to the species *Mus musculus*, C57BL/6J strain. All procedures involving animal models were approved by the Animal Use Ethics Committee (CEUA) of the University of Campinas (UNICAMP) – São Paulo, Brazil (6391–1/2024). Six animals were transplanted in each experimental group. In both groups, deaths occurred, with one death recorded in the treated group and two in the control group. In the treated group, one animal showed a high level of chimerism (~95%), while another presented a very low level and was therefore excluded from the analysis. Thus, three animals with intermediate levels of chimerism (mean approximately 75%) were included in the analysis. In the control group, animals with chimerism levels comparable to those observed in the treated group were selected, totaling three animals for analysis..

To obtain the donor cells, a sickle cell mouse was anesthetized with a combination of ketamine (100 mg/kg) and xylazine (10 mg/kg), ensuring adequate sedation, analgesia, and muscle relaxation for the procedure. For euthanasia, a high anesthetic dose (300 mg/kg ketamine and 30 mg/kg xylazine) was administered, followed by cervical dislocation, in accordance with the guidelines of the Animal Ethics Committee (CEUA) and CONCEA. In addition, measures were implemented throughout the entire experiment to minimize animal suffering, including continuous monitoring, appropriate anesthesia and analgesia protocols, and procedures conducted by a trained team, in compliance with the 3Rs principles. After euthanasia, the long bones (femur and tibia) from the hind limbs were collected. Following disinfection with 70% ethanol, the bones were washed with DMEM medium, which was also used for bone marrow extraction using insulin syringes. The cell suspension was homogenized and subjected to red blood cell lysis using ammonium-chloride-potassium (ACK) solution in order to isolate hematopoietic progenitor cells.

The recipient mice were previously subjected to irradiation with a dose of 900 rads of gamma radiation to eliminate bone marrow cells and allow for the integration of the transplanted cells. The donor cell infusion was performed intravenously at a dose of 3 × 10⁶ cells in 0,1 mL, using the orbital plexus as the administration route—an effective and less invasive method widely used in experimental models.

The choice of the transplantation methodology was due to the limited availability and fragility of established transgenic SCD models, which restrict their application in large-scale experimental studies. Moreover, the transplanted model allows for the evaluation of specific pathophysiological aspects and the testing of therapeutic interventions in a controlled manner, contributing to the advancement of research on the disease

### Haptoglobin cloning using the pGEM-T Easy vector

The cloning of the haptoglobin protein was performed using recombinant DNA technology. The cDNA corresponding to the messenger RNA (mRNA) of human haptoglobin was obtained by reverse transcription followed by PCR (RT-PCR), using specific primers designed on the Benchling platform. The reaction was carried out in a final volume of 25 µL, containing 12.5 µL of CloneAmp mix (including enzyme, buffer, and dNTPs), 1 µL of each primer, 5 µL of cDNA, and 5.5 µL of water.

The amplified fragment was inserted into the pGEM-T Easy vector (Promega), which has 3’ thymine overhangs, facilitating ligation with PCR-generated products. The ligation reaction was carried out in a 10 µL volume containing 5 µL of T4 DNA ligase buffer, 1 µL of the vector, 3 µL of the PCR product, and 1 µL of T4 DNA ligase enzyme, incubated overnight at 4 °C.

After ligation, the recombinant vector was transformed into competent *Escherichia coli* cells, which were cultured in selective medium containing ampicillin. The resulting colonies were screened by PCR to identify clones containing the insert.

Subsequently, the selected coding fragments were subcloned into the Lego tdTomato vector, aiming at gene expression of haptoglobin with the tdTomato reporter gene assisted by the P2A cleavage peptide.

### Cloning of haptoglobin into the Lego-tdTomato plasmid

After the initial cloning of the haptoglobin coding sequence into the pGEM-T Easy vector, the region of interest was subcloned into the lego tdTomato expression plasmid, derived from pEGFP-C1. Unlike the original vector, this plasmid expresses the red fluorescent protein tdTomato, allowing the tracking of transfected cells through fluorescence microscopy. The expression vector was designed for bicistronic expression of haptoglobin, concomitant with the tdTomato reporter protein separated by the P2A cleavage peptide. Expression control was carried out by the SFFV (Spleen Focus-Forming Virus) promoter, known to drive strong gene expression, to promote overexpression of the haptoglobin transgene. The human haptoglobin sequence (GenBank: NG_012651.1) was inserted into the lego tdTomato plasmid using restriction enzymes and T4 DNA ligase.

After confirmation of cloning through analysis of bacterial growth, the recombinant vector containing haptoglobin was subjected to sequencing and was also introduced into HEK 293-T cells by lipid-based transfection to verify gene expression. These cells, derived from human embryonic kidney, were chosen because they are widely used in gene expression experiments due to their high transcription and translation rates, as well as their ease of culture and handling.

The cells were cultured until they reached appropriate confluence for transfection. The expression of the tdTomato protein was evaluated by fluorescence microscopy, with red fluorescence emission indicating successful expression of the construct containing both proteins. This step allowed the selection of transfected cells and the validation of the gene expression system for subsequent phases of the experiment.

### Sequencing

Genetic sequencing was used to validate the results obtained by PCR and to confirm the integrity of the sequences of interest. The technique employed was Sanger sequencing, which is based on the extension of DNA fragments from specific primers, with the incorporation of deoxynucleotide triphosphates (dNTPs), generating products of different sizes that allow the reading of the nucleotide sequence.

Recombinant plasmids were extracted using the QIAprep Spin Miniprep kit (QIAGEN) in order to eliminate impurities such as residual dNTPs, free primers, enzymes, and salts. Quantification and purity assessment of the samples were performed by spectrophotometry using a nanodrop, considering absorbance at 260 nm as an indicator of nucleic acid concentration.

For sequencing, specific oligonucleotides targeting the regions of interest were designed using the Benchling platform. Data interpretation was conducted based on the analysis of chromatograms generated by the sequencing service, using Chromas software.

### Introduction of the plasmid into animals

Assays were conducted for the transfection of the Hapto-tdTomato plasmid. The efficiency of gene expression was determined based on the fluorescence observed in hepatic cells after transfection. The plasmid was prepared for animal transfection via lipid-based transfection. Ten micrograms (10 µg) of plasmid were mixed with the commercial reagent JetOPTIMUS solution according to the manufacturer’s instructions, and administered to mouse models transplanted one week earlier with sickle hematopoietic progenitor cells, via the retro-orbital plexus with 100 µL of the solution. The animals were euthanized after 60 days for analysis of gene expression by microscopy to verify the expression of the tdTomato fluorescent protein in the hepatic parenchyma, as well as for tissue collection for pathological analysis.

### Pathological analysis

To evaluate the effects of the proposed treatment in a sickle cell anemia experimental model, comparative pathological analyses were conducted, in a double- blind manner, between control and treated groups. The methodological approaches included quantification of hemoglobins by HPLC, complete blood count, assessment of spleen weight, and histological analysis of target organs. These evaluations enabled the investigation of hematological, morphological, and tissue alterations associated with the disease and the treatment. Data were analyzed using GraphPad Prism software, applying the unpaired t-test with a significance level of 5%. Results were presented in graphs and expressed as mean ± standard deviation.

1. High-performance liquid chromatography

The qualitative and quantitative determination of hemoglobin fractions in peripheral blood was performed by cation-exchange high-performance liquid chromatography (HPLC), using the automated Variant II system (Bio-Rad). Engraftment was confirmed using this approach. Peripheral blood samples were collected via submandibular puncture and automatically aspirated, injected into the analytical system, and applied to a cation-exchange column (stationary phase). A programmed buffer gradient (mobile phase), driven by dual-piston pumps, increased the ionic strength and promoted the separation of different hemoglobin fractions based on their ionic interactions with the column. The eluted fractions were detected by photometry at 415 nm, identified by their characteristic retention times, and quantified by the area under the elution peaks.

2. Complete Blood Count (CBC)

The complete blood count was performed on anticoagulated whole blood samples using the automated hematology analyzer XN-9000 (Sysmex). This system employs four detection methods: the RF/DC (radiofrequency/direct current) method, which evaluates cell volume through direct current resistance and intracellular density based on variations in radiofrequency resistance; hydrodynamic focusing, which ensures cell alignment in the laminar flow, minimizing artifacts and false impulses during counting; flow cytometry with a semiconductor laser, used to analyze blood cells and particles through light emission by stained RNA, DNA, and proteins, allowing the separation of cell populations using scatter plot analysis; and the SLS-hemoglobin method, which uses sodium lauryl sulfate (SLS), free of cyanide, to lyse erythrocytes and form SLS-HGB complexes, whose concentration is determined by photometry using a monochromatic LED and detection by a photosensor.

3. Spleen weight

For the analysis of spleen weight, the animals were euthanized according to a protocol approved by the ethics committee, and the spleen was carefully dissected, removed, and immediately weighed on a precision analytical balance. The data obtained were used for comparison between the experimental groups, considering both the absolute weight of the organ and its ratio to the total body weight of the animals. This analysis aimed to evaluate splenic alterations associated with the sickle cell condition and the effects of the proposed treatment.

4. **Histopathological assessment**

For histological analysis, the animals were euthanized according to a protocol approved by the ethics committee. The organs of interest were collected, fixed in 10% buffered formalin solution, routinely processed, embedded in paraffin, and sectioned using a microtome at a thickness of 4 μm. The slides were stained with hematoxylin and eosin (HE) and analyzed under a light microscope. Histological evaluation was performed comparatively between groups, considering morphological parameters and the presence of tissue alterations associated with sickle cell anemia and the effects of the experimental treatment.

## Results

**Fig 1:**
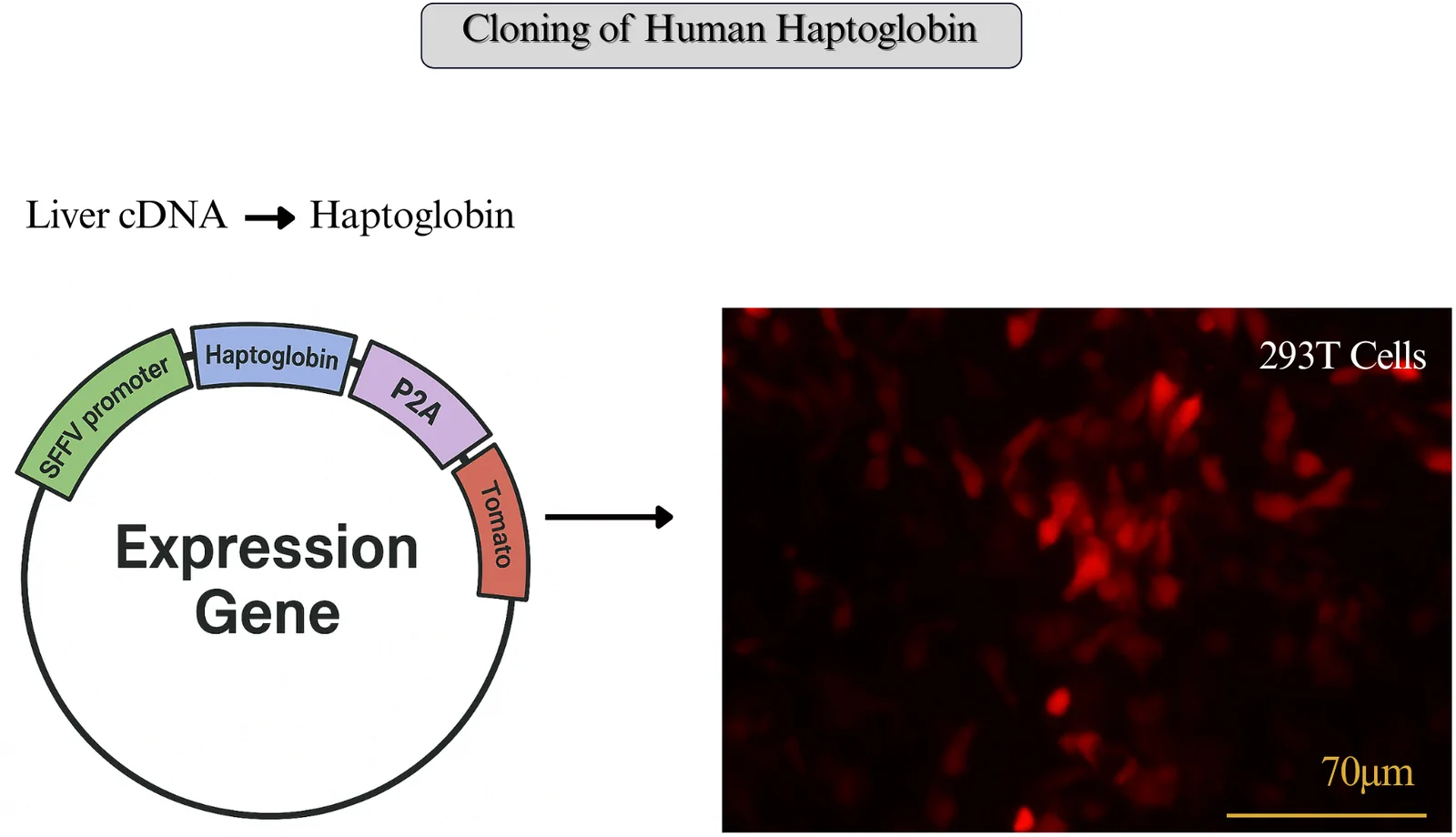
Schematic representation of haptoglobin expression vector construction and validation. The plasmid contains the SFFV promoter, the human haptoglobin coding sequence, the P2A cleavage site, and the td-Tomato fluorescent marker, allowing bicistronic expression and visualization of transgene expression in 293-T cells.

To investigate the contribution of haptoglobin overexpression in an organism with chronic hemolysis during the development of sickle cell anemia, we initially constructed a strong expression vector to express haptoglobin and a reporter protein to facilitate its tracking (Fig 1). The vector is driven by the strong promoter of the Spleen Focus-Forming Virus – SFFV, producing a single messenger RNA with bicistronic expression, first of the haptoglobin protein and second of the TdTomato reporter gene (red fluorescent) in frame. This ensures efficient expression and visualization of gene expression in cells. The SFFV promoter is known for its strong constitutive activity, ensuring robust and stable expression. Next, the coding sequence of human haptoglobin was inserted from a hepatic cDNA library. To allow simultaneous but independent expression of a fluorescent marker, the P2A cleavage peptide was used, which promotes ribosomal skipping during translation, allowing the production of two separate proteins from a single transcript. The chosen marker was tdTomato, a fluorescent gene derived from DsRed, which emits intense and stable red fluorescence. The presence of tdTomato in the construct allows for the visual identification and tracking of cells expressing the haptoglobin gene, facilitating the selection and functional analysis of transfectants. 293-T cells were used to validate the construct expression system.

**Fig 2:**
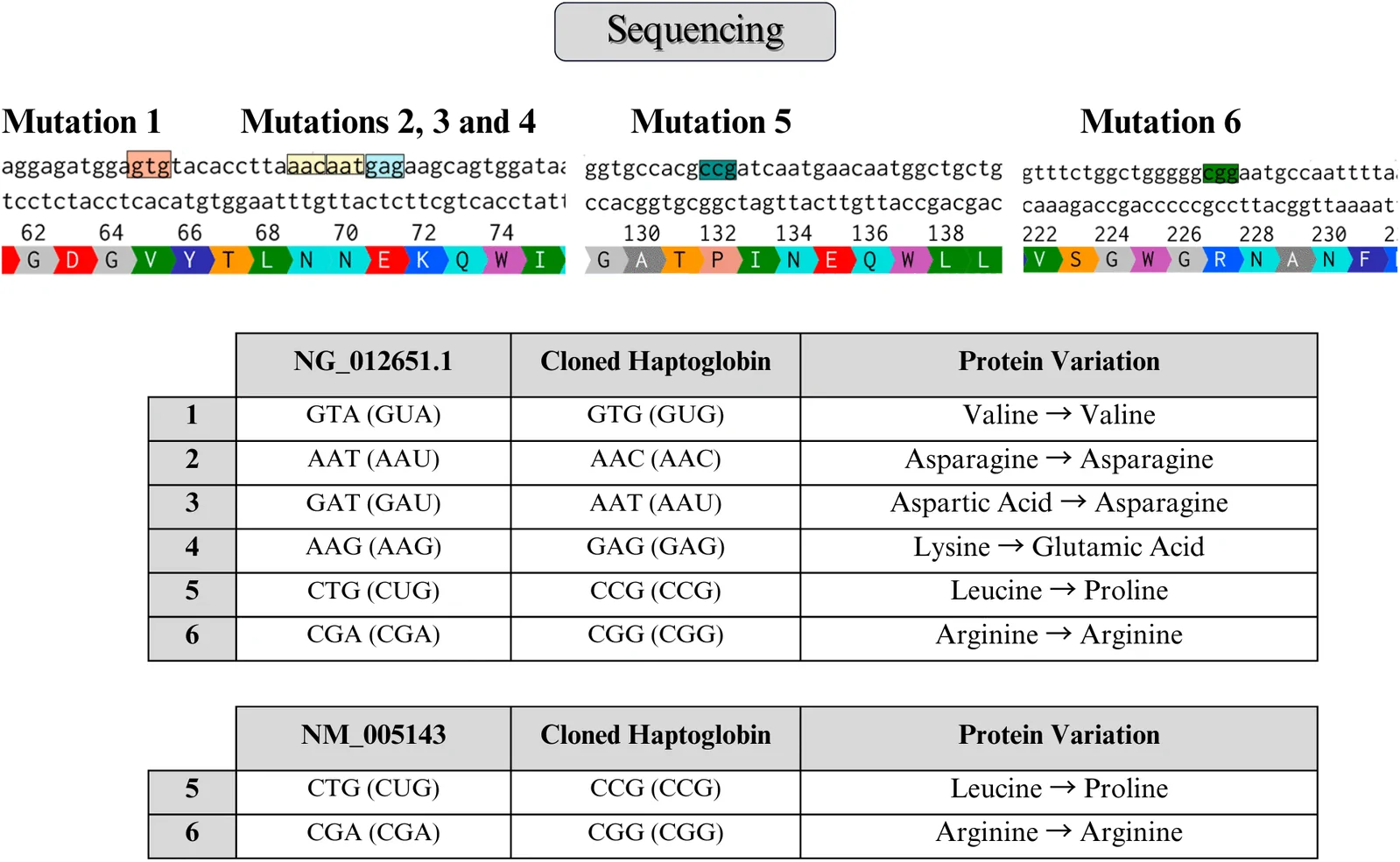
Sequencing analysis of the cloned haptoglobin gene. Alignment with the reference sequences NM_005143 (gene) and NP_005134 (protein) revealed 99.58% and 99.37% identity, respectively.

To ensure greater fidelity of the haptoglobin sequence, the sequence cloned into the vector was sequenced (Fig 2). The sequence showed 99,74% identity with the reference gene sequence NG_012651.1; 99.79% with the mRNA sequence NM_005143 using the BLASTN tool, and 99.69% with the reference protein sequence NP_005134 using BLASTP. Comparison with the reference sequence revealed high identity, with only six-point variations, three of which resulted in amino acid substitutions. The observed changes included: substitution of aspartic acid with asparagine, which may impact electrostatic interactions and the protein’s catalytic activity; replacement of lysine with glutamic acid, altering the charge profile of the region and potentially influencing interactions with substrates or other biomolecules; and substitution of leucine with proline, a change that may affect the local conformation of the polypeptide chain due to proline’s structural rigidity. Despite these modifications, the obtained sequence maintains high similarity to the reference, and the point mutations do not appear to compromise the expected functionality of the protein.

**Fig 3:**
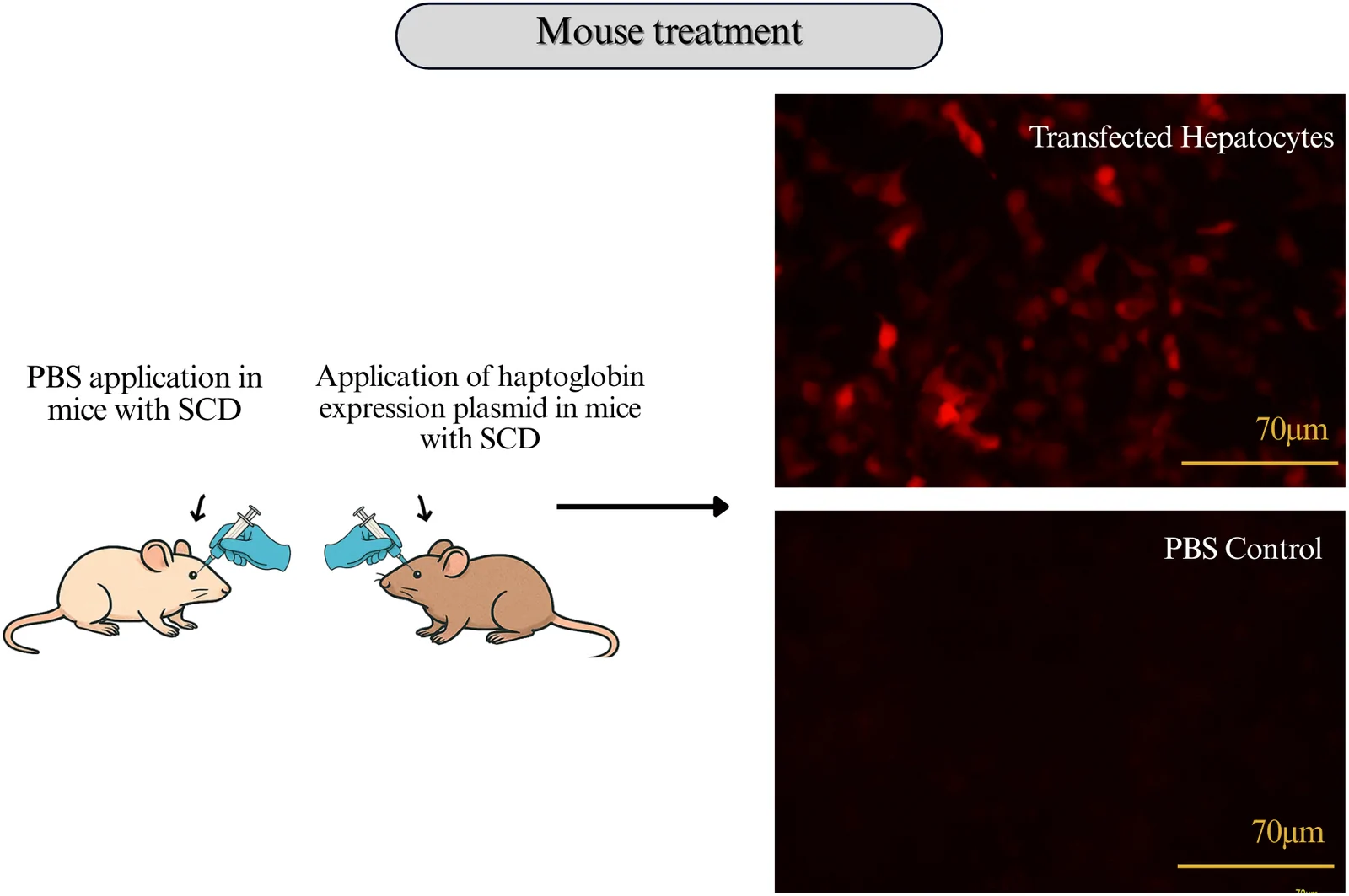
Plasmid delivery and hepatic expression in SCD mice (n = 3). Hepatic red fluorescence observed 26 days after retro-orbital injection of the hapto-tdTomato plasmid confirms efficient delivery and expression.

**Fig 4:**
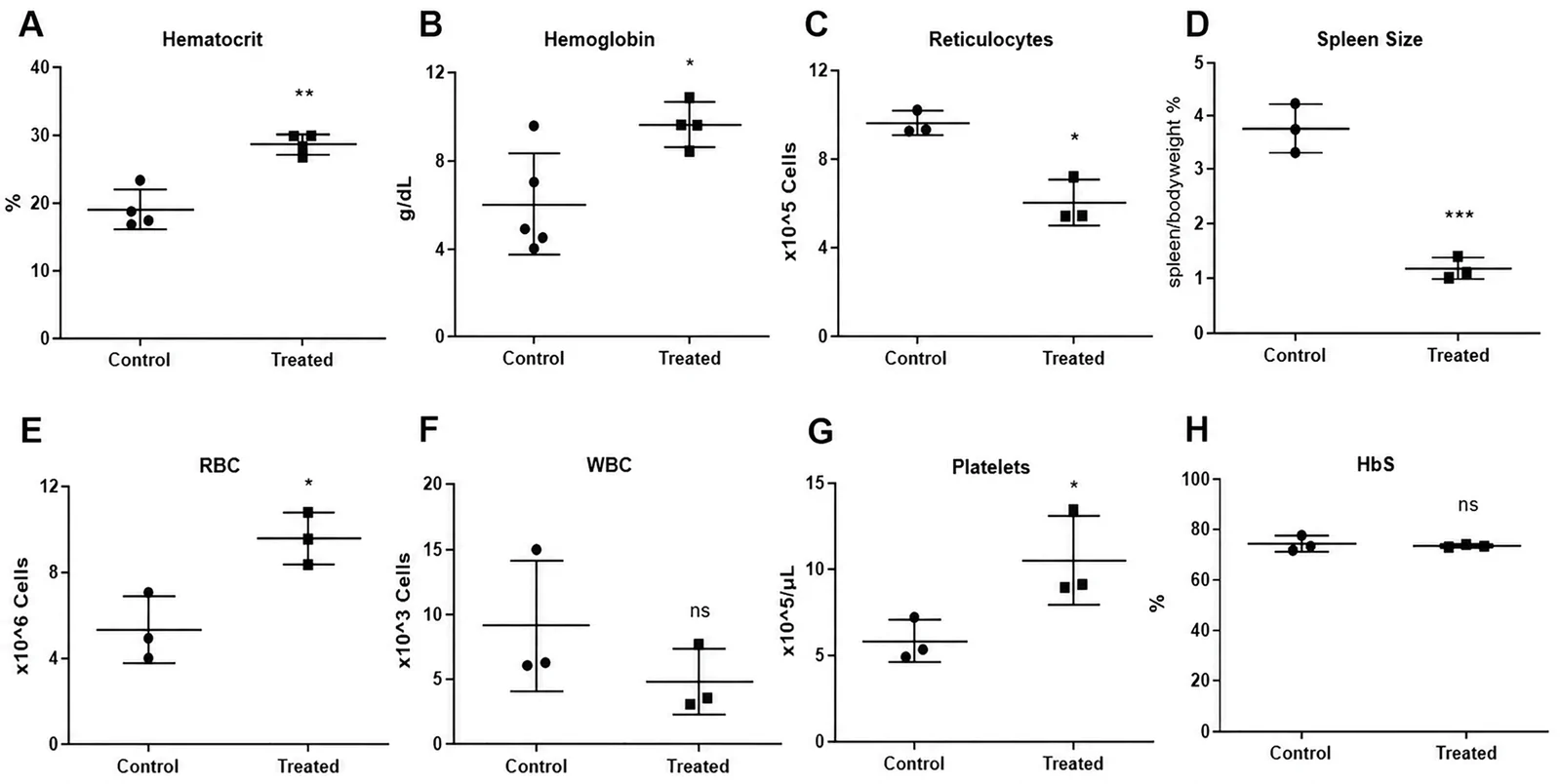
Hematological parameters and spleen size in SCD mice after haptoglobin plasmid treatment (n = 3). Treatment increased hematocrit and hemoglobin levels, reduced reticulocyte counts, and decreased relative spleen size compared to untreated SCD mice. In addition, treated animals showed increased red blood cell (RBC) counts and platelet levels, while white blood cell (WBC) counts and %HbS remained unchanged. Data are presented as mean ± SD. Statistical significance is indicated as * (p < 0.05), ** (p < 0.01), *** (p < 0.001), and ns (not significant).

After the *in vitro* analyses, the application of the plasmid was evaluated in a mouse model of sickle cell anemia. The methodology adopted had been previously standardized using cationic liposomes for hepatic transfection of the plasmid via intravenous administration through the orbital plexus with 100 µL of buffered saline solution containing 10 µL of liposomes and 10 µg of the construct. After 26 days, microscopy revealed red fluorescence in multiple hepatic regions (Fig 3), confirming the efficacy of the protocol. The 26-day time point for the analyses was selected based on logistical aspects related to experimental organization and equipment availability. The plasmid containing the cloned sequence was introduced into animals developing SCD one week after transplantation, and PBS was used in animals assigned as SHAM. After 60 days of disease progression, the animals were sacrificed, and physiological samples of blood and organs such as liver, spleen, and kidney were collected for analysis of clinical parameters to validate the treatment. Among the analyzed parameters, hemoglobin levels, hematocrit, and reticulocytes stand out, as they are important indicators of the animals’ hematological status (Fig 4).

Hematological analysis (Fig 4) revealed deleterious and expected alterations in animals with SCD, which were partially protected by treatment with haptoglobin transfection. The hematocrit of SCD animals ranged from 16.80 to 23.45%, indicating severe anemia. In the treated group, the values increased significantly (26.70 to 30.10%; p = 0.0013), although still below the physiological range for mice (35–50%), undoubtedly suggesting improved stability and/or production of erythrocytes. Hemoglobin levels also increased with treatment (8.50–11.0 g/dL) compared to the SCD group (4.60–9.70 g/dL), with a significant difference (p = 0.0236), lifting the animals out of a severe anemia condition. Due to limited blood volume, free hemoglobin and plasma haptoglobin could not be reliably quantified, as haptoglobin levels were below the detection limit of the assay.. Following this physiological improvement profile, the reticulocyte count, elevated in the SCA group (9.225–10.193 × 10^5^ cells), was reduced in the treated group (5.408–7.205 × 10^5^ cells), with a statistically significant difference (p = 0.0015). This reduction suggests a decrease in compensatory erythropoiesis, reflecting improved erythrocyte survival.

Platelet counts were also significantly elevated in the treated group, indicating a partial recovery of hematopoietic function, especially in myeloid lineage. In contrast, white blood cell (WBC) counts did not differ significantly between groups, with high inter-sample variability observed. Differential leukocyte counts were not performed in this study, limiting a more detailed assessment of specific inflammatory cell populations such as neutrophils. Similarly, no significant changes were observed in %HbS levels between treated and untreated groups, indicating that the therapeutic effects observed are likely related to modulation of hemolysis rather than alterations in hemoglobin composition. Finally, a significant lower size in the relative spleen size (weight/body weight) was observed in the treated animals (p = 0.0009), suggesting decreased sequestration and erythrocyte destruction. The SCD group showed splenomegaly, characteristic of chronic hemolysis, whereas the treatment protected the organism from spleen enlargement, indicating an attenuation of the functional overload (Fig 4).

**Fig 5:**
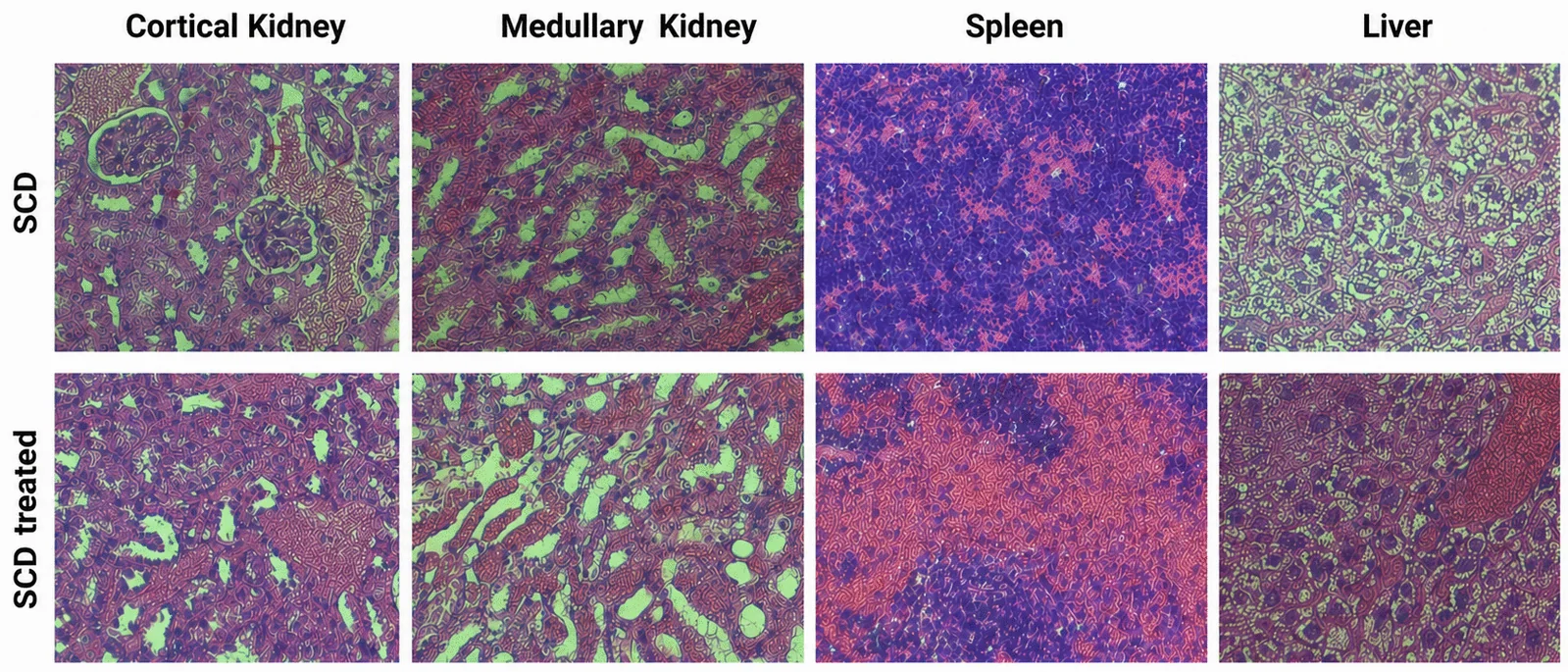
Histological changes in kidney, spleen, and liver of SCD mice after haptoglobin plasmid treatment (n = 3). Kidneys from untreated SCD mice showed red blood cell accumulation, edema, and vascular congestion; treated mice showed partial structural preservation. Spleens from untreated mice presented white pulp disorganization and intense inflammation; treated mice showed improved architecture. Livers from untreated mice displayed sinusoidal dilation, inflammatory infiltration, and deformed red blood cell accumulation; treated mice had better sinusoid organization and signs of reduced hepatic stress.

Complementarily, histological evaluation revealed significant tissue impairment in the target organs of sickle cell anemia, with partial impact from the treatment. In the kidneys of animals with SCD, red blood cell accumulation, edema, and vascular dilation were observed, especially in the cortical region, along with congestion in the medullary region. These alterations reflect microvascular obstruction induced by sickling of red blood cells. In the treated group, although the presence of red blood cells was still evident, the renal structures did not show worsening, indicating a possible partial containment of the damage (Fig 5).

Hepatic histology of animals with SCD revealed sinusoidal dilation, inflammatory infiltration, and accumulation of deformed red blood cells, compromising intrahepatic circulation. In treated animals, despite the persistence of red blood cells in the vessels, better organization of the sinusoids was observed, along with indications of reduced hepatic stress.

These histopathological findings confirm the systemic impact of sickle cell anemia on multiple organs and indicate that the tested treatment was able to promote partial improvements in inflammatory and structural aspects, especially in the spleen and liver, although it was insufficient to fully reverse the alterations associated with the sickle cell condition.

## Discussion

Chronic hemolysis in Sickle Cell Disease leads to the persistent accumulation of extracellular and intravascular free hemoglobin, promoting endothelial dysfunction, oxidative stress, and disturbances in vascular homeostasis that contribute directly to the clinical manifestations of the disease. In the present study, we focused on haptoglobin, the major endogenous scavenger of circulating free hemoglobin, whose physiological buffering capacity becomes overwhelmed under chronic hemolytic conditions such as sickle cell disease. To further get evidence of the protective role of haptoglobin in this context, we induced the in vivo hepatic expression of haptoglobin-1 in a murine model developing sickle cell disease generated through bone marrow transplantation using the Berkeley SCD model. By increasing circulating haptoglobin levels and favoring the formation of Hb–Hp complexes, we investigated whether enhanced hemoglobin clearance could attenuate the deleterious consequences associated with chronic hemolysis.

Transgenic models have been widely used to mimic human diseases as sickle cell disease [16]. The Berkely SCD model used in this work reproduces relevant phenotypic characteristics of SCD, including alterations in red blood cell morphology and impaired tissue oxygenation, and are also useful for evaluating therapeutic interventions [15,17].

We performed irradiated murine models subjected to SCD bone marrow transplantation. The irradiation eliminates the recipient’s hematopoietic cells, promoting the integration of donor-derived SCD model’s stem cells [14]. Following transplantation, hematological, molecular, and functional parameters are evaluated.

The transplanted models were considered to exhibit severe sickle cell anemia phenotype 60 days post-transplantation, with an HbS proportion above 70%, as described by Iannone et al [18]. Wild-type red blood cells, gamma-irradiated erythrocytes exhibit a significantly reduced lifespan, estimated at approximately 10 days. Therefore, at 60 days post-transplantation, animals predominantly present erythrocytes derived from the transplanted bone marrow, expressing physiological and molecular features consistent with sickle cell anemia, regarding animals with more than 60% of HbS post transplanted [18]. Additionally, in the absence of transplantation, animals do not survive beyond 7–10 days due to the lack of functional erythrocytes.

Regarding the treatment of SCD, several approaches have been proposed to improve patients’ clinical outcomes. Hydroxyurea remains the standard therapy, demonstrating efficacy in reducing complications across different age groups, although its clinical acceptance remains limited, which drives the search for alternative strategies [19,20]. In this context, novel therapeutic targets include inflammation, cell adhesion, hemolysis, and endothelial dysfunction, and new drugs have been approved, while numerous clinical trials are investigating agents with complementary mechanisms [19]. Therapies such as L-glutamine, voxelotor, and crizanlizumab have shown benefits by reducing oxidative stress, improving hemoglobin oxygen affinity, and decreasing vaso-occlusive events [19]. In parallel, more advanced strategies include hematopoietic stem cell transplantation and gene therapy, with gene addition and gene editing approaches capable of correcting mutations or reactivating fetal hemoglobin production, the latter being considered a promising long-term curative alternative [21].

In this context, haptoglobin (Hp) has been widely investigated due to its role in binding and clearing free hemoglobin, thereby contributing to the reduction of hemolysis-associated toxicity. Studies indicate that increased Hp levels can neutralize circulating toxic components and prevent complications such as albuminuria [22]. In addition, Hp has been proposed as a biomarker in conditions such as transplant-associated thrombotic microangiopathy, having been identified as a promising protein in proteomic analyses [23]. Experimental evidence demonstrates that Hp can reduce vascular injury, vaso-occlusive crises, and hemoglobin accumulation in tissues [24].The Hp–Hb complex acts as a scavenger of free hemoglobin and heme, reducing oxidative stress and inflammation [25]. This complex is taken up by macrophages via the CD163 receptor, activating heme oxygenase-1 and promoting cytoprotective and anti-inflammatory effects [7]. Furthermore, Hp plays a relevant role in preventing vascular damage associated with chronic hemolysis [26],as well as in immune modulation, renal protection, and its association with various clinical conditions [27]. Despite all these characteristics, the high level of haptoglobin sequestration in a chronic hemolytic disease may affect conclusive responses in the organism. Therefore, to get insights regarding Hp effects into the chronic hemolytic background, we have analyzed a mouse model on SCD formation with continuous extra expression of Hp by gene therapy in hepatic tissue. Although previous studies did not observe good transgene expression in hepatic tissue using cationic liposome solution inoculated via the tail vein of rats [28], it appears that the orbital plexus route was effective in our case. Contrary to the results obtained by those authors, we did not observe transgene expression in the lung and spleen, but rather effective expression in hepatic tissue. These results indicate that efficient plasmid delivery and marker expression were achieved, establishing a solid methodological foundation for our objectives.

Our results obtained in this study partially corroborate the literature, demonstrating beneficial effects associated with Hp. Although its action is primarily described as occurring after hemolysis, improvements in hemolytic status and increased erythrocyte resistance were observed, possibly through indirect mechanisms related to the reduction of the toxic effects of free hemoglobin. Interestingly, haptoglobin acts after hemolysis by clearing free hemoglobin from the organism; however, our results indicate an improvement in the hemolytic condition and increased erythrocyte resistance. To date, there is no evidence of a direct effect of haptoglobin in reducing hemolysis, and the observed results may be due to an indirect action—through the reduction of the harmful effects of free hemoglobin in an organism with chronic hemolysis. Our findings are consistent with a study that reported very similar results to ours, showing improvements in organ anatomy as well as hematological parameters in beta-thalassemic mice treated with an apohemoglobin–haptoglobin complex [25]. Like them, we believe that the reduction or control of the release of harmful factors from hemolysis into the bloodstream, even in organisms with chronic hemolysis—such as in sickle cell and thalassemic conditions—can lead to a decrease in factors like cytokines and enzymes that worsen the inflammatory process and oxidative stress. This may favor physiological hemolysis, rather than being solely attributed to the genetic deficiency of red blood cells. Although we did not conduct in-depth studies on inflammation, our results may suggest this theory. Another study also in thalassemia demonstrated the slowing of arteriosclerosis as an indirect effect of hemopexin treatment [13]. In addition to these observations, the extended hematological analysis provided further insight into the effects of haptoglobin treatment. Treated animals exhibited increased red blood cell (RBC) counts, reinforcing the observed improvements in hematocrit and hemoglobin levels and supporting enhanced erythrocyte survival and/or reduced hemolysis. Platelet counts were also significantly elevated, suggesting a partial recovery of hematopoietic function and a possible reduction of inflammatory process, although this finding should be interpreted with caution given the complex role of platelets in SCD pathophysiology.

In contrast, no significant changes were observed in total leukocyte counts, which showed considerable variability among samples. Differential leukocyte analysis, including neutrophil counts, was not performed, limiting a more detailed evaluation of inflammatory responses. Similarly, %HbS levels remained unchanged between groups, indicating that the therapeutic effects of haptoglobin are likely associated with modulation of hemolysis and its downstream consequences rather than direct alterations in hemoglobin composition.

In line with these observations, despite our study could not precisely determine the anti-hemolytic effect mediated by haptoglobin overexpression, the work by Hurtado et al. (2023) suggests indirect beneficial effects on the attenuation of inflammatory and vascular processes in experimental models of hemolytic diseases treated with hemopexin [26]. A more comprehensive analysis of inflammatory markers would be required to better elucidate the underlying mechanism.

Overall, the plasmid construct containing the human Hp gene demonstrated efficient in vivo expression and promoted relevant therapeutic effects in the SCD model, including hematological improvement, reduction of compensatory erythropoiesis, and attenuation of clinical signs such as splenomegaly. Partial preservation of organ structure was also observed, particularly in the liver and spleen, although without complete reversal of microvascular alterations, reinforcing the potential of recombinant Hp as a therapeutic strategy and highlighting the need for further optimization of this approach.

Histological analysis in murine models proved to be essential for understanding the cellular and tissue-level effects of the interventions, allowing the identification of structural, inflammatory, and regenerative changes. In SCD, marked alterations are observed mainly in the spleen, liver, and kidneys. In the spleen, autosplenectomy may occur as a result of repeated infarctions caused by vascular obstruction, leading to fibrosis, atrophy, and impaired immune function [29], whereas in healthy conditions the splenic architecture remains preserved, with proper organization of the white and red pulp [30]. In the liver, sinusoidal dilation and inflammatory infiltration associated with chronic hemolysis are observed, while in the kidneys there is inflammation, edema, and microvascular congestion, compromising renal function [31]. These findings reinforce the systemic effects of SCD and highlight the impact of continuous hemolysis on multiple organs.

A pioneering study evaluating repeated administration of synthetic haptoglobin (Hp) in a murine model of sickle cell disease (Townes mice) demonstrated modest therapeutic effects on disease-associated organ damage [32]. Administration of high doses of synthetic Hp (400 mg/kg) increased the expression of heme oxygenase-1 (HO-1) and ferritin heavy chain (ferritin-H), while reducing iron deposition in the kidneys. In addition, a trend toward a reduced incidence of hepatic infarction was observed. Although this latter finding is consistent with our observations, the remaining outcomes differ substantially, as the therapeutic strategy employed in the present study produced broader and more pronounced biological effects.

In our experimental model, sustained Hp overexpression induced by gene therapy resulted in significant histological improvement of the spleen, together with a consistent recovery of erythroid hematological parameters, as evidenced by the attenuation of anemia and the overall improvement of the hematological indices previously described and discussed. These findings suggest that continuous Hp expression exerted protective effects not only against tissue injury but also on the hematological pathophysiology that characterizes sickle cell disease.

The differences observed between the two studies are likely attributable to differences in both the experimental model and the therapeutic strategy employed. Whereas the previous study was based on the intermittent administration of synthetic Hp in Townes mice, our model was established by transplantation of bone marrow from Berkeley mice with severe sickle cell disease into lethally irradiated C57BL/6 recipient mice, generating a stable and severe hematological phenotype approximately 60 days after transplantation. Therapeutic intervention was initiated at this stage, when the pathological process was already established and the initial manifestations of organ injury were developing.

In this context, sustained Hp expression appears to have primarily delayed or attenuated the progression of hemolysis-associated comorbidities rather than completely preventing their development. These findings reinforce the importance of modulating the Hp-free hemoglobin (Hb) scavenging pathway to mitigate the progressive tissue damage associated with sickle cell disease, while also indicating that this strategy alone is not sufficient to fully halt disease progression.

The efficacy of this mechanism is likely dependent on the balance between the amount of available Hp and the continuous burden of free hemoglobin released during chronic intravascular hemolysis. In sickle cell disease, endogenous Hp production rapidly becomes insufficient to neutralize the high concentrations of extracellular Hb, resulting in saturation of the physiological Hp-Hb scavenging system. From this perspective, gene therapy approaches capable of promoting sustained, high-level Hp expression may represent a promising therapeutic strategy, particularly when combined with the initial administration of exogenous recombinant Hp. Such a combined approach would enable the rapid neutralization of circulating free Hb present at the onset of treatment, while continuous Hp expression driven by gene therapy would progressively restore the capacity for extracellular Hb clearance, thereby re-establishing vascular homeostasis and reducing the deleterious consequences of chronic hemolysis.

The interpretation of the present findings should consider experimental limitations inherent to studies involving severe sickle cell disease (SCD) animal models. The generation and maintenance of these models are technically demanding due to their low reproductive performance, high susceptibility to experimental manipulation, and reduced survival, all of which substantially limit sample availability. These challenges are further compounded in bone marrow transplantation-based models, in which achieving homogeneous levels of sickle hemoglobin (HbS) expression among recipient animals represents an additional source of biological variability.

To minimize this variability, only animals exhibiting the highest degree of similarity in HbS levels were included in the experimental groups, thereby increasing the consistency and internal validity of the analyses despite the relatively small sample size. Although this approach inevitably reduced the number of animals available for statistical analysis, it strengthened the biological comparability between groups and increased the reliability of the observed treatment effects.

Another important limitation was the restricted volume of blood that could be collected from animals presenting with severe anemia, which constrained the number of complementary analyses that could be performed. Consequently, additional investigations, including comprehensive inflammatory profiling, longitudinal quantification of circulating cell-free hemoglobin before and after treatment, and pharmacokinetic assessment of circulating Hp concentrations throughout the treatment period, could not be performed. Such analyses would provide valuable mechanistic insights into the protective effects observed in the present study.

The continuous intravascular hemolysis characteristic of SCD, together with the rapid formation and subsequent clearance of Hp-Hb complexes, likely resulted in rapid consumption of the administered Hp, leaving circulating concentrations below the detection limit of the analytical method employed. Therefore, more sensitive analytical approaches will be required in future studies to accurately characterize Hp pharmacokinetics and to establish the relationship between circulating Hp levels, free Hb scavenging capacity, and therapeutic efficacy. Such investigations will be essential for optimizing Hp-based therapeutic strategies and further elucidating the mechanisms underlying the beneficial effects observed in the present study.

Furthermore, the results obtained open perspectives for future investigations, including the improvement of haptoglobin-based therapeutic approaches, the development of combined strategies, and the expansion of diagnostic and therapeutic applications in hemolytic diseases, reinforcing the importance of continuing this line of research.
