# Supplementary material for: Therapeutic potential of haptoglobin in a murine model of sickle cell anemia
Source: PLoS One. 2026 Sep 18;21(9):e0357180. doi: 10.1371/journal.pone.0357180 (PMC13588521; doi:10.1371/journal.pone.0357180)
Supplement: Supplementary file 1 [file pone.0357180.s002.docx]

We would like to thank the reviewers for their careful evaluation of our manuscript, as well as for their valuable comments and suggestions. We sincerely appreciate the time and effort dedicated to the assessment of our work.

All comments were carefully considered, and the manuscript has been revised accordingly. The changes made are highlighted in the revised version, and detailed responses to each point raised are provided below.

We believe that these revisions have significantly improved the clarity and overall quality of the manuscript.

**Editor’s Comment:**

Please include the following items when submitting your revised manuscript: a response letter, a marked-up version of the manuscript, and a clean version of the manuscript.
**Authors’ Response:**

All requested documents have been properly prepared and submitted according to the instructions: (i) the response letter to the reviewers, (ii) the revised manuscript with tracked changes, and (iii) the final clean version of the manuscript without markings.

**Editor’s Comment:**

If you wish to make changes to your financial disclosure, please include the updated version in your cover letter. We also recommend depositing your laboratory protocols in protocols.io.

**Authors’ Response:**

The cover letter has been reviewed and updated as necessary. Regarding the recommendation to make protocols/data available, we inform that these have been deposited in the institutional repository of the University of Campinas (REDU), with the corresponding identifier: <https://doi.org/10.25824/redu/KYLRCN>

**Editor’s Comment:**

Please ensure that your manuscript meets the PLOS ONE style requirements, including file naming conventions.

**Authors’ Response:**

The manuscript has been reviewed and adjusted to comply with PLOS ONE style requirements, including formatting and file naming, as instructed.

**Editor’s Comment:**

Provide additional information in the Methods section regarding animal experiments, including euthanasia methods, anesthesia/analgesia, and efforts to alleviate suffering.

**Author Response:**

We thank the reviewer for this comment. The requested information has been included and detailed in the Methods section of the revised manuscript.
Briefly, animals were anesthetized with a combination of ketamine (100 mg/kg) and xylazine (10 mg/kg), ensuring adequate sedation, analgesia, and muscle relaxation for the experimental procedures. For euthanasia, a high anesthetic dose (300 mg/kg ketamine and 30 mg/kg xylazine) was administered, followed by cervical dislocation, in accordance with the guidelines of the Animal Ethics Committee (CEUA) and CONCEA.
In addition, measures were implemented throughout the entire experiment to minimize animal suffering, including continuous monitoring, appropriate anesthesia and analgesia protocols, and procedures conducted by a trained team, in compliance with the 3Rs principles.

**Editor’s Comment:**

A revised Funding Statement is requested, including all sources of support, as well as the inclusion of the sentence “No additional external funding was received for this study.”

**Author Response:**

We thank the reviewer for this comment. The Funding Statement has been revised and updated to include all sources of support received during the conduct of the study, in accordance with the journal guidelines. The requested sentence (“No additional external funding was received for this study”) has also been included. The updated Funding Statement has been incorporated into the cover letter.

**Editor’s Comment:**

Please indicate the role of the funders in the study, including the appropriate statement in the cover letter.

**Author Response:**

We thank the reviewer for this comment. The statement regarding the role of the funders has been included in the cover letter, as requested. Specifically, we state that the funders had no role in study design, data collection and analysis, decision to publish, or preparation of the manuscript.

**Editor’s Comment:**

Please remove funding information from the Acknowledgements section and update the Funding Statement.

**Author Response:**

We thank the reviewer for this comment. The funding-related information has been removed from the Acknowledgements section of the manuscript, as instructed.
The Funding Statement has been revised and updated in accordance with the journal guidelines, and the corrected version has been included in the cover letter for the corresponding updates in the submission system.

**Editor’s Comment:**

Please confirm and, if necessary, update the Data Availability Statement, ensuring access to the minimum dataset required to replicate the study.

**Author Response:**

We thank the reviewer for this comment. The Data Availability Statement has been revised to ensure compliance with the PLOS ONE guidelines.
The minimum dataset required for replication of the study results has been made publicly available in an institutional repository (REDU – University of Campinas), with the corresponding link/DOI: <https://doi.org/10.25824/redu/KYLRCN>
In addition, we confirm that all data relevant to the interpretation of the results are included within the manuscript, the supplementary files, and/or the indicated repository.

**Editor’s Comment:**

Figure 3 contains copyrighted images. Please obtain permission or replace the figure with content compliant with the CC BY 4.0 license.

**Author Response:**

We thank the reviewer for this comment. Figure 3 has been replaced with a new original version, without the use of any previously copyrighted material, and is fully compliant with the CC BY 4.0 license requirements.

**Reviewer Comment:**

What is the number of mice used? [...] In addition, the graphs could be presented as individual points instead of bar graphs.

**Author Response:**

We thank the reviewer for this comment. Information regarding the number of animals has been included and detailed in the revised manuscript.
Six animals were transplanted in each experimental group. In both groups, deaths occurred, with one death recorded in the treated group and two in the control group. In the treated group, one animal showed a high level of chimerism (~95%), while another presented a very low level and was therefore excluded from the analysis. Thus, three animals with intermediate levels of chimerism (mean approximately 75%) were included in the analysis.
In the control group, animals with chimerism levels comparable to those observed in the treated group were selected, totaling three animals for analysis.
Additionally, the number of animals (n = 3) has been included in the figure legends. We consider that this information allows appropriate interpretation of the presented results.

**Reviewer Comment:**

Why was haptoglobin 1 overexpression chosen instead of haptoglobin 2? Please justify this choice and discuss the clinical implications in the context of Hp1 versus Hp2 comparison.

**Author Response:**

We thank the reviewer for this comment. The choice of haptoglobin phenotype Hp1 was based on literature evidence demonstrating the greater efficiency of this phenotype in neutralizing the deleterious effects of free hemoglobin.
Studies indicate that Hp1-1 exhibits higher antioxidant capacity compared to Hp2-2, promoting the formation of more stable Hb-Hp complexes with more efficient clearance via endocytosis. In contrast, complexes formed with Hp2-2 are associated with increased generation of reactive oxygen species, thereby enhancing the potential for oxidative damage to lipids, proteins, and DNA.
Therefore, considering the context of chronic hemolysis observed in sickle cell anemia, Hp1 was considered more appropriate as a therapeutic strategy due to its greater efficiency in reducing oxidative stress and its potential tissue-protective effects (LEVY et al., 2010; PIRES; BIERHALZ; MORAES, 2015; PIRES; BERTHIAUME; PALMER, 2023; QUIMBY; HAMBLETON; LANDIS, 2015).
The clinical implications of this choice have been included and further discussed in the revised version of the manuscript.

**Reviewer Comment:**

Describe how and when the SS bone marrow graft was confirmed, which method was used, and discuss the impact of circulating wild-type red blood cells at the time of treatment.

**Author Response:**

We thank the reviewer for this comment. The engraftment was confirmed by high-performance liquid chromatography (HPLC) using the Variant II automated system (Bio-Rad). Peripheral blood samples were collected via submandibular puncture (cheek pouch), subsequently aspirated, injected into the analytical system, and applied to a cation-exchange column. A programmed buffer gradient enabled the separation of different hemoglobin fractions based on their ionic interactions with the column. Eluted fractions were detected by photometry at 415 nm, identified by their characteristic retention times, and quantified by peak area integration.
The transplanted models were considered to exhibit a severe sickle cell anemia phenotype 60 days post-transplantation, with an HbS proportion above 70%, as described by Iannone et al. (2001).
Regarding the presence of wild-type red blood cells, it is important to note that gamma-irradiated erythrocytes exhibit a significantly reduced lifespan, estimated at approximately 10 days, as reported in the literature. Therefore, at 60 days post-transplantation, animals predominantly present erythrocytes derived from the transplanted bone marrow, expressing physiological and molecular features consistent with sickle cell anemia. Additionally, in the absence of transplantation, animals do not survive beyond 7–10 days due to the lack of functional erythrocytes.
 These points have been included and discussed in the revised manuscript.

**Reviewer Comment:**

Indicate whether inflammatory parameters (e.g., leukocytes, neutrophils) were assessed after treatment and discuss their relevance to the therapeutic potential of haptoglobin.

**Author Response:**

We thank the reviewer for this comment. Differential leukocyte counts were not performed in the present study. Regarding total leukocyte counts, high variability among samples was observed, and no statistically significant differences were identified between groups.

On the other hand, a significant increase in platelet counts was observed in the treated animals.

We emphasize that a more precise evaluation of the inflammatory profile would require the assessment of specific markers, such as cytokines, which were not performed in this study; however, this approach will be considered in future investigations.

Nevertheless, as described in the literature (Hurtado et al., 2023), hemolytic conditions are associated with increased levels of free heme, which can promote oxidative stress and inflammatory responses through activation of innate immune pathways. Therefore, by reducing circulating free hemoglobin and its downstream heme-related toxicity, haptoglobin may indirectly contribute to the modulation of inflammatory processes.

**Reviewer Comment:**

Minor corrections in the text, including conceptual adjustments, addition of references, citation standardization, methodological detailing, and figure corrections (lines 24, 53, 59, 62–71, 65, 76, 95, 102, 173, 301, 312, and 313).

**Author Response:**

We thank the reviewer for these comments. All minor corrections have been addressed in the revised manuscript, including the removal and adjustment of terms, addition and correction of references, standardization of citations, further methodological detailing, and correction of inconsistencies in both the figures and the text.
In addition, the requested information, such as reference ranges and the mentioned hematological parameters, has been included.

**Reviewer Comment:**

Although genetic variability was included, the model name was not mentioned. Are the bone marrow donor animals from the Townes or Berkeley model?

**Author Response:**

We thank the reviewer for this comment. The bone marrow donor mice used in this study belong to the Berkeley model, a widely used humanized model of sickle cell disease that predominantly expresses human hemoglobin S.
Additionally, the irradiated recipient animals belong to the species *Mus musculus*, C57BL/6J strain.
This information has been included in the revised manuscript.

**Reviewer Comment:**

The manuscript presents several issues related to the discussion of the results, which is predominantly descriptive. It is recommended to expand the discussion by incorporating critical analysis and comparison with previous studies in the literature.

**Author Response:**

The Discussion section has been revised and expanded, including comparisons with previous studies in the literature, in order to provide a more in-depth critical analysis of the obtained results.

**Reviewer Comment:**

Why was the 26-day time point chosen for analysis? In addition, please include an image of the PBS SS control in Figure 3.

**Author Response:**

We thank the reviewer for this comment. The 26-day time point for the analyses was selected based on logistical aspects related to experimental organization and equipment availability. Nevertheless, this period proved to be appropriate for the manifestation of the evaluated changes, as demonstrated by the results obtained in the study.
Additionally, as suggested, the image corresponding to the PBS SS control was included in the revised figure, allowing for a better comparison between the experimental groups.

**Reviewer Comment:**

Erythrocyte production is suggested; why was the number of erythrocytes not included in Figure 4?

**Author Response:**

We thank the reviewer for this comment. The erythrocyte count has been included in the revised version of Figure 4, as suggested.

**Reviewer Comment:**

Did you attempt to measure free hemoglobin? In addition, did plasma haptoglobin levels change?

**Author Response:**

We thank the reviewer for this comment. The amount of biological material available (blood) was limited, which restricted the performance of additional analyses, including the measurement of free hemoglobin.
Regarding plasma haptoglobin, attempts were made to quantify it; however, the values obtained did not reach the minimum detection limit of the equipment used. Considering the context of sickle cell anemia, in which there is marked consumption of haptoglobin due to its binding to free hemoglobin, a detectable level of this parameter was not expected.
Therefore, it was not possible to obtain reliable measurements for these markers in the present study.

**Closing Remarks**

We would like to thank the Editor and the reviewers once again for their careful evaluation of the manuscript and for the valuable suggestions provided. All comments were carefully considered, and we believe that the revisions made have significantly improved the clarity, consistency, and scientific quality of the work.

We hope that the revised version meets the expectations, and we remain available for any further clarifications that may be required.
