# Supplementary material for: Therapeutic potential of haptoglobin in a murine model of sickle cell anemia
Source: PLoS One. 2026 Sep 18;21(9):e0357180. doi: 10.1371/journal.pone.0357180 (PMC13588521; doi:10.1371/journal.pone.0357180)
Supplement: Peer review history

# Peer review: Decision Letter 0

**DOI:** 10.1371/journal.pone.0357180.r001

**Authors:** Fábio Silva

13 Oct 2025

Dear Dr. Corat,

Thank you for submitting your manuscript to PLOS ONE. After careful consideration, we feel that it has merit but does not fully meet PLOS ONE’s publication criteria as it currently stands. Therefore, we invite you to submit a revised version of the manuscript that addresses the points raised during the review process.

Please submit your revised manuscript by Nov 27 2025 11:59PM. If you will need more time than this to complete your revisions, please reply to this message or contact the journal office at plosone@plos.org. When you're ready to submit your revision, log on to https://www.editorialmanager.com/pone/ and select the 'Submissions Needing Revision' folder to locate your manuscript file.

- A rebuttal letter that responds to each point raised by the academic editor and reviewer(s). You should upload this letter as a separate file labeled 'Response to Reviewers'.
- A marked-up copy of your manuscript that highlights changes made to the original version. You should upload this as a separate file labeled 'Revised Manuscript with Track Changes'.
- An unmarked version of your revised paper without tracked changes. You should upload this as a separate file labeled 'Manuscript'.

If you would like to make changes to your financial disclosure, please include your updated statement in your cover letter. Guidelines for resubmitting your figure files are available below the reviewer comments at the end of this letter.

If applicable, we recommend that you deposit your laboratory protocols in protocols.io to enhance the reproducibility of your results. Protocols.io assigns your protocol its own identifier (DOI) so that it can be cited independently in the future. For instructions see: https://journals.plos.org/plosone/s/submission-guidelines#loc-laboratory-protocols. Additionally, PLOS ONE offers an option for publishing peer-reviewed Lab Protocol articles, which describe protocols hosted on protocols.io. Read more information on sharing protocols at https://plos.org/protocols?utm_medium=editorial-email&utm_source=authorletters&utm_campaign=protocols.

We look forward to receiving your revised manuscript.

Kind regards,

Fábio Henrique Silva, Ph.D.

Academic Editor

PLOS ONE

Journal Requirements:

When submitting your revision, we need you to address these additional requirements.

1. Please ensure that your manuscript meets PLOS ONE's style requirements, including those for file naming. The PLOS ONE style templates can be found at

https://journals.plos.org/plosone/s/file?id=wjVg/PLOSOne_formatting_sample_main_body.pdf and

https://journals.plos.org/plosone/s/file?id=ba62/PLOSOne_formatting_sample_title_authors_affiliations.pdf

2. To comply with PLOS One submissions requirements, in your Methods section, please provide additional information regarding the experiments involving animals and ensure you have included details on (1) methods of sacrifice, (2) methods of anesthesia and/or analgesia, and (3) efforts to alleviate suffering.

3. Thank you for stating in your Funding Statement:

“São Paulo Research Foundation (FAPESP) for supporting project nº 2019/18886-1, and the Coordination for the Improvement of Higher Education Personnel (CAPES) project nº 88887.676144/2022-00”

Please provide an amended statement that declares *all* the funding or sources of support (whether external or internal to your organization) received during this study, as detailed online in our guide for authors at http://journals.plos.org/plosone/s/submit-now. Please also include the statement “There was no additional external funding received for this study.” in your updated Funding Statement.

Please include your amended Funding Statement within your cover letter. We will change the online submission form on your behalf.

4. Thank you for stating the following financial disclosure:

“São Paulo Research Foundation (FAPESP) for supporting project nº 2019/18886-1, and the Coordination for the Improvement of Higher Education Personnel (CAPES) project nº 88887.676144/2022-00”

Please state what role the funders took in the study. If the funders had no role, please state: "The funders had no role in study design, data collection and analysis, decision to publish, or preparation of the manuscript."

If this statement is not correct you must amend it as needed.

Please include this amended Role of Funder statement in your cover letter; we will change the online submission form on your behalf.

5. Thank you for stating the following in the Acknowledgments Section of your manuscript:

“We would like to thank the São Paulo Research Foundation (FAPESP) for supporting project nº 2019/18886-1, and the Coordination for the Improvement of Higher Education Personnel (CAPES) project nº 88887.676144/2022-00, linked to the Ministry of Education of Brazil.”

We note that you have provided funding information that is currently declared in your Funding Statement. However, funding information should not appear in the Acknowledgments section or other areas of your manuscript. We will only publish funding information present in the Funding Statement section of the online submission form.

Please remove any funding-related text from the manuscript and let us know how you would like to update your Funding Statement. Currently, your Funding Statement reads as follows:

“São Paulo Research Foundation (FAPESP) for supporting project nº 2019/18886-1, and the Coordination for the Improvement of Higher Education Personnel (CAPES) project nº 88887.676144/2022-00”

Please include your amended statements within your cover letter; we will change the online submission form on your behalf.

6. We note that your Data Availability Statement is currently as follows: All relevant data are within the manuscript and its Supporting Information files.

Please confirm at this time whether or not your submission contains all raw data required to replicate the results of your study. Authors must share the “minimal data set” for their submission. PLOS defines the minimal data set to consist of the data required to replicate all study findings reported in the article, as well as related metadata and methods (https://journals.plos.org/plosone/s/data-availability#loc-minimal-data-set-definition).

For example, authors should submit the following data:

- The values behind the means, standard deviations and other measures reported;

- The values used to build graphs;

- The points extracted from images for analysis.

Authors do not need to submit their entire data set if only a portion of the data was used in the reported study.

If your submission does not contain these data, please either upload them as Supporting Information files or deposit them to a stable, public repository and provide us with the relevant URLs, DOIs, or accession numbers. For a list of recommended repositories, please see https://journals.plos.org/plosone/s/recommended-repositories.

If there are ethical or legal restrictions on sharing a de-identified data set, please explain them in detail (e.g., data contain potentially sensitive information, data are owned by a third-party organization, etc.) and who has imposed them (e.g., an ethics committee). Please also provide contact information for a data access committee, ethics committee, or other institutional body to which data requests may be sent. If data are owned by a third party, please indicate how others may request data access.

7. We note that Figure 3 in your submission contain copyrighted images. All PLOS content is published under the Creative Commons Attribution License (CC BY 4.0), which means that the manuscript, images, and Supporting Information files will be freely available online, and any third party is permitted to access, download, copy, distribute, and use these materials in any way, even commercially, with proper attribution. For more information, see our copyright guidelines: http://journals.plos.org/plosone/s/licenses-and-copyright.

We require you to either (1) present written permission from the copyright holder to publish these figures specifically under the CC BY 4.0 license, or (2) remove the figures from your submission:

a. You may seek permission from the original copyright holder of Figure 3 to publish the content specifically under the CC BY 4.0 license.

We recommend that you contact the original copyright holder with the Content Permission Form (http://journals.plos.org/plosone/s/file?id=7c09/content-permission-form.pdf) and the following text:

“I request permission for the open-access journal PLOS ONE to publish XXX under the Creative Commons Attribution License (CCAL) CC BY 4.0 (http://creativecommons.org/licenses/by/4.0/). Please be aware that this license allows unrestricted use and distribution, even commercially, by third parties. Please reply and provide explicit written permission to publish XXX under a CC BY license and complete the attached form.”

Please upload the completed Content Permission Form or other proof of granted permissions as an "Other" file with your submission.

In the figure caption of the copyrighted figure, please include the following text: “Reprinted from [ref] under a CC BY license, with permission from [name of publisher], original copyright [original copyright year].”

b. If you are unable to obtain permission from the original copyright holder to publish these figures under the CC BY 4.0 license or if the copyright holder’s requirements are incompatible with the CC BY 4.0 license, please either i) remove the figure or ii) supply a replacement figure that complies with the CC BY 4.0 license. Please check copyright information on all replacement figures and update the figure caption with source information. If applicable, please specify in the figure caption text when a figure is similar but not identical to the original image and is therefore for illustrative purposes only.

8. If the reviewer comments include a recommendation to cite specific previously published works, please review and evaluate these publications to determine whether they are relevant and should be cited. There is no requirement to cite these works unless the editor has indicated otherwise.

Additional Editor Comments:

Dear Dr. Corat,

Thank you for submitting your manuscript entitled “Therapeutic potential of haptoglobin in a murine model of sickle cell anemia” to PLOS ONE.

The study addresses a relevant and timely topic and has potential translational impact. However, one of the reviewers raised several major methodological and reporting concerns that must be addressed before the manuscript can be reconsidered for publication.

Key points to address in your revision include:

1. Report the number of animals used in each group (total transplanted, excluded, final N), and present individual data points in the figures rather than only bar graphs.

2. Provide a justification for the choice of haptoglobin 1, and discuss the clinical implications of this decision in the context of Hp1 versus Hp2.

3. Describe in detail how and when SS bone marrow engraftment was confirmed, and discuss the potential impact of circulating wild-type red blood cells at the time of treatment.

4. Address whether inflammatory parameters (e.g., leukocytes, neutrophils) were evaluated after treatment, and discuss their relevance to the therapeutic potential of haptoglobin.

5. Add missing references, clearly state the model used, anesthetic protocols, and software employed for analysis.

6. Expand the Discussion section. We recommend separating “Results” and “Discussion” into two distinct sections to improve clarity, structure, and scientific depth. The discussion should compare and contrast your findings with relevant previous studies that have investigated haptoglobin treatment in SCD models, highlighting advances and limitations.

7. Revise the textual issues and figure labeling inconsistencies noted by the reviewer (e.g., figure numbering, references, terminology).

8. Given the significance of these points, the editorial decision is Major Revision. Please submit a revised version of your manuscript with a detailed, point-by-point response to each reviewer comment.

We look forward to receiving your revised manuscript.

Sincerely,

Dr. Fabio Henrique Silva

Academic Editor

PLOS ONE

[Note: HTML markup is below. Please do not edit.]

Reviewers' comments:

Reviewer's Responses to Questions

**Comments to the Author**

1. Is the manuscript technically sound, and do the data support the conclusions?

Reviewer #1: No

Reviewer #2: Yes

**********

2. Has the statistical analysis been performed appropriately and rigorously?

Reviewer #1: N/A

Reviewer #2: Yes

**********

3. Have the authors made all data underlying the findings in their manuscript fully available?

The PLOS Data policy

Reviewer #1: No

Reviewer #2: Yes

**********

4. Is the manuscript presented in an intelligible fashion and written in standard English?

Reviewer #1: Yes

Reviewer #2: Yes

**********

Reviewer #1: General questions:

1-What is the number of mice used? At no point did I see how many mice were transplanted, how many had the SS bone marrow implanted, or how many were discarded. The N of mice in the figures are also not included. Furthermore, the graphs could be plotted as points for each number, rather than a closed bar.

2-Why did you choose overexpression of haptoglobin 1 and not 2? Or both for have the same human phenotype.

3- Knowing that mouse red blood cells have a lifespan of 40-60 days, healthy red blood cells continue to circulate for this period after transplantation. Did you confirm SS marrow engraftment by hemoglobin electrophoresis/liquid chromatography? Or what method?

In line 196 it looks like to have been done by chromatography, but it is not clear how this sample was collected and at what point post-transplant.

Furthermore, in line 181, you said that the treatment was carried out 1 week after the transplant, so you treated the animals without knowing if the SS marrow had engaged, and even if it had switched to SS, the 'wild' red blood cells continued to circulate until approximately the date of sacrifice, which was 60 days after the treatment. Would this be a limitation of study?

4- Considering Haptoglobin as a possible treatment for sickle cells, after your treatment, did you see any decrease in inflammatory parameters? such as leukocytes and especially neutrophils? In the blood count?

__________

Line 24 - where you say -the genetic, you can remove it, continue- with sickle cell disease...

Line 53 – Need add reference.

Line 59 - A reference from 1969 was cited in the text, in the references look like a thesis from 2008, please cite the original reference.

Line 65 - blood components, such as renal cells, if they are renal cells are not blood components.

The text from line 62 to 71 needs to be better referenced.

Line 76 – Need add reference.

Line 94 - Although you included the genetic variety, they didn't include the model’s name. Are these MO donor animals Tim Townes or Berkeley? This makes it easier for the reader.

Line 95 - Include only the first author, do not include all of them.

Line 102- Include the concentration and/or % and proportion of the anesthetics.

Line 108 - What species are these mice that were irradiated and received the bone marrow?

Line 173 - which software?

Lines 239 to 276 – only described the results and did not discuss them with previous work. I think the entire work needs to be better discussed; most of the results and discussion sections are just describing the results and not discussing them.

Line 287-296-297 -why did you choose 26 days? And not before or after? Also, including Figure 3, do you have an image of the PBS control SS? I think it would be important to show the difference in images.

Line 301- Fig 3 when in fact it is figure 4.

Line 312 - Reference normal range.

Line 313- Suggests RBC production, why was the number of RBCs not included in Figure 4?

Line 320 - Did you try to measure free hemoglobin? Additionally, did the plasma haptoglobin levels change?

Reviewer #2: The study is relevant and presents a promising gene therapy strategy using haptoglobin in a murine model of sickle cell anemia. The data are consistent with the conclusions, and the work makes a significant contribution to the field of experimental therapies.

Some suggestions are to provide the raw data (individual values) and to clearly report the number of animals (n) in each experimental group.

**********

what does this mean?). If published, this will include your full peer review and any attached files.

If you choose “no”, your identity will remain anonymous but your review may still be made public.

**Do you want your identity to be public for this peer review?**  For information about this choice, including consent withdrawal, please see our Privacy Policy

Reviewer #1: No

Reviewer #2: **Yes:**  Tammyris Helena Rebecchi e Silveira

**********

[NOTE: If reviewer comments were submitted as an attachment file, they will be attached to this email and accessible via the submission site. Please log into your account, locate the manuscript record, and check for the action link "View Attachments". If this link does not appear, there are no attachment files.]

While revising your submission, please upload your figure files to the Preflight Analysis and Conversion Engine (PACE) digital diagnostic tool, https://pacev2.apexcovantage.com/. PACE helps ensure that figures meet PLOS requirements. To use PACE, you must first register as a user. Registration is free. Then, login and navigate to the UPLOAD tab, where you will find detailed instructions on how to use the tool. If you encounter any issues or have any questions when using PACE, please email PLOS at figures@plos.org. Please note that Supporting Information files do not need this step.

# Author comment: Author response to Decision Letter 1

**DOI:** 10.1371/journal.pone.0357180.r002

27 May 2026

We would like to thank the reviewers for their careful evaluation of our manuscript, as well as for their valuable comments and suggestions. We sincerely appreciate the time and effort dedicated to the assessment of our work.

All comments were carefully considered, and the manuscript has been revised accordingly. The changes made are highlighted in the revised version, and detailed responses to each point raised are provided below.

We believe that these revisions have significantly improved the clarity and overall quality of the manuscript.

________________________________________

Editor’s Comment:

Please include the following items when submitting your revised manuscript: a response letter, a marked-up version of the manuscript, and a clean version of the manuscript.

Authors’ Response:

All requested documents have been properly prepared and submitted according to the instructions: (i) the response letter to the reviewers, (ii) the revised manuscript with tracked changes, and (iii) the final clean version of the manuscript without markings.

Editor’s Comment:

If you wish to make changes to your financial disclosure, please include the updated version in your cover letter. We also recommend depositing your laboratory protocols in protocols.io.

Authors’ Response:

The cover letter has been reviewed and updated as necessary. Regarding the recommendation to make protocols/data available, we inform that these have been deposited in the institutional repository of the University of Campinas (REDU), with the corresponding identifier: https://doi.org/10.25824/redu/KYLRCN

Editor’s Comment:

Please ensure that your manuscript meets the PLOS ONE style requirements, including file naming conventions.

Authors’ Response:

The manuscript has been reviewed and adjusted to comply with PLOS ONE style requirements, including formatting and file naming, as instructed.

Editor’s Comment:

Provide additional information in the Methods section regarding animal experiments, including euthanasia methods, anesthesia/analgesia, and efforts to alleviate suffering.

Author Response:

We thank the reviewer for this comment. The requested information has been included and detailed in the Methods section of the revised manuscript.

Briefly, animals were anesthetized with a combination of ketamine (100 mg/kg) and xylazine (10 mg/kg), ensuring adequate sedation, analgesia, and muscle relaxation for the experimental procedures. For euthanasia, a high anesthetic dose (300 mg/kg ketamine and 30 mg/kg xylazine) was administered, followed by cervical dislocation, in accordance with the guidelines of the Animal Ethics Committee (CEUA) and CONCEA.

In addition, measures were implemented throughout the entire experiment to minimize animal suffering, including continuous monitoring, appropriate anesthesia and analgesia protocols, and procedures conducted by a trained team, in compliance with the 3Rs principles.

Editor’s Comment:

A revised Funding Statement is requested, including all sources of support, as well as the inclusion of the sentence “No additional external funding was received for this study.”

Author Response:

We thank the reviewer for this comment. The Funding Statement has been revised and updated to include all sources of support received during the conduct of the study, in accordance with the journal guidelines. The requested sentence (“No additional external funding was received for this study”) has also been included. The updated Funding Statement has been incorporated into the cover letter.

Editor’s Comment:

Please indicate the role of the funders in the study, including the appropriate statement in the cover letter.

Author Response:

We thank the reviewer for this comment. The statement regarding the role of the funders has been included in the cover letter, as requested. Specifically, we state that the funders had no role in study design, data collection and analysis, decision to publish, or preparation of the manuscript.

Editor’s Comment:

Please remove funding information from the Acknowledgements section and update the Funding Statement.

Author Response:

We thank the reviewer for this comment. The funding-related information has been removed from the Acknowledgements section of the manuscript, as instructed.

The Funding Statement has been revised and updated in accordance with the journal guidelines, and the corrected version has been included in the cover letter for the corresponding updates in the submission system.

Editor’s Comment:

Please confirm and, if necessary, update the Data Availability Statement, ensuring access to the minimum dataset required to replicate the study.

Author Response:

We thank the reviewer for this comment. The Data Availability Statement has been revised to ensure compliance with the PLOS ONE guidelines.

The minimum dataset required for replication of the study results has been made publicly available in an institutional repository (REDU – University of Campinas), with the corresponding link/DOI: https://doi.org/10.25824/redu/KYLRCN

In addition, we confirm that all data relevant to the interpretation of the results are included within the manuscript, the supplementary files, and/or the indicated repository.

Editor’s Comment:

Figure 3 contains copyrighted images. Please obtain permission or replace the figure with content compliant with the CC BY 4.0 license.

Author Response:

We thank the reviewer for this comment. Figure 3 has been replaced with a new original version, without the use of any previously copyrighted material, and is fully compliant with the CC BY 4.0 license requirements.

Reviewer Comment:

What is the number of mice used? [...] In addition, the graphs could be presented as individual points instead of bar graphs.

Author Response:

We thank the reviewer for this comment. Information regarding the number of animals has been included and detailed in the revised manuscript.

Six animals were transplanted in each experimental group. In both groups, deaths occurred, with one death recorded in the treated group and two in the control group. In the treated group, one animal showed a high level of chimerism (~95%), while another presented a very low level and was therefore excluded from the analysis. Thus, three animals with intermediate levels of chimerism (mean approximately 75%) were included in the analysis.

In the control group, animals with chimerism levels comparable to those observed in the treated group were selected, totaling three animals for analysis.

Additionally, the number of animals (n = 3) has been included in the figure legends. We consider that this information allows appropriate interpretation of the presented results.

Reviewer Comment:

Why was haptoglobin 1 overexpression chosen instead of haptoglobin 2? Please justify this choice and discuss the clinical implications in the context of Hp1 versus Hp2 comparison.

Author Response:

We thank the reviewer for this comment. The choice of haptoglobin phenotype Hp1 was based on literature evidence demonstrating the greater efficiency of this phenotype in neutralizing the deleterious effects of free hemoglobin.

Studies indicate that Hp1-1 exhibits higher antioxidant capacity compared to Hp2-2, promoting the formation of more stable Hb-Hp complexes with more efficient clearance via endocytosis. In contrast, complexes formed with Hp2-2 are associated with increased generation of reactive oxygen species, thereby enhancing the potential for oxidative damage to lipids, proteins, and DNA.

Therefore, considering the context of chronic hemolysis observed in sickle cell anemia, Hp1 was considered more appropriate as a therapeutic strategy due to its greater efficiency in reducing oxidative stress and its potential tissue-protective effects (LEVY et al., 2010; PIRES; BIERHALZ; MORAES, 2015; PIRES; BERTHIAUME; PALMER, 2023; QUIMBY; HAMBLETON; LANDIS, 2015).

The clinical implications of this choice have been included and further discussed in the revised version of the manuscript.

Reviewer Comment:

Describe how and when the SS bone marrow graft was confirmed, which method was used, and discuss the impact of circulating wild-type red blood cells at the time of treatment.

Author Response:

We thank the reviewer for this comment. The engraftment was confirmed by high-performance liquid chromatography (HPLC) using the Variant II automated system (Bio-Rad). Peripheral blood samples were collected via submandibular puncture (cheek pouch), subsequently aspirated, injected into the analytical system, and applied to a cation-exchange column. A programmed buffer gradient enabled the separation of different hemoglobin fractions based on their ionic interactions with the column. Eluted fractions were detected by photometry at 415 nm, identified by their characteristic retention times, and quantified by peak area integration.

The transplanted models were considered to exhibit a severe sickle cell anemia phenotype 60 days post-transplantation, with an HbS proportion above 70%, as described by Iannone et al. (2001).

Regarding the presence of wild-type red blood cells, it is important to note that gamma-irradiated erythrocytes exhibit a significantly reduced lifespan, estimated at approximately 10 days, as reported in the literature. Therefore, at 60 days post-transplantation, animals predominantly present erythrocytes derived from the transplanted bone marrow, expressing physiological and molecular features consistent with sickle cell anemia. Additionally, in the absence of transplantation, animals do not survive beyond 7–10 days due to the lack of functional erythrocytes.

These points have been included and discussed in the revised manuscript.

Reviewer Comment:

Indicate whether inflammatory parameters (e.g., leukocytes, neutrophils) were assessed after treatment and discuss their relevance to the therapeutic potential of haptoglobin.

Author Response:

We thank the reviewer for this comment. Differential leukocyte counts were not performed in the present study. Regarding total leukocyte counts, high variability among samples was observed, and no statistically significant differences were identified between groups.

On the other hand, a significant increase in platelet counts was observed in the treated animals.

We emphasize that a more precise evaluation of the inflammatory profile would require the assessment of specific markers, such as cytokines, which were not performed in this study; however, this approach will be considered in future investigations.

Nevertheless, as described in the literature (Hurtado et al., 2023), hemolytic conditions are associated with increased levels of free heme, which can promote oxidative stress and inflammatory responses through activation of innate immune pathways. Therefore, by reducing circulating free hemoglobin and its downstream heme-related toxicity, haptoglobin may indirectly contribute to the modulation of inflammatory processes.

Reviewer Comment:

Minor corrections in the text, including conceptual adjustments, addition of references, citation standardization, methodological detailing, and figure corrections (lines 24, 53, 59, 62–71, 65, 76, 95, 102, 173, 301, 312, and 313).

Author Response:

We thank the reviewer for these comments. All minor corrections have been addressed in the revised manuscript, including the removal and adjustment of terms, addition and correction of references, standardization of citations, further methodological detailing, and correction of inconsistencies in both the figures and the text.

In addition, the requested information, such as reference ranges and the mentioned hematological parameters, has been included.

Reviewer Comment:

Although genetic variability was included, the model name was not mentioned. Are the bone marrow donor animals from the Townes or Berkeley model?

Author Response:

We thank the reviewer for this comment. The bone marrow donor mice used in this study belong to the Berkeley model, a widely used humanized model of sickle cell disease that predominantly expresses human hemoglobin S.

Additionally, the irradiated recipient animals belong to the species Mus musculus, C57BL/6J strain.

This information has been included in the revised manuscript.

Reviewer Comment:

The manuscript presents several issues related to the discussion of the results, which is predominantly descriptive. It is recommended to expand the discussion by incorporating critical analysis and comparison with previous studies in the literature.

Author Response:

The Discussion section has been revised and expanded, including comparisons with previous studies in the literature, in order to provide a more in-depth critical analysis of the obtained results.

Reviewer Comment:

Why was the 26-day time point chosen for analysis? In addition, please include an image of the PBS SS control in Figure 3.

Author Response:

We thank the reviewer for this comment. The 26-day time point for the analyses was selected based on logistical aspects related to experimental organization and equipment availability. Nevertheless, this period proved to be appropriate for the manifestation of the evaluated changes, as demonstrated by the results obtained in the study.

Additionally, as suggested, the image corresponding to the PBS SS control was included in the revised figure, allowing for a better comparison between the experimental groups.

Reviewer Comment:

Erythrocyte production is suggested; why was the number of erythrocytes not included in Figure 4?

Author Response:

We thank the reviewer for this comment. The erythrocyte count has been included in the revised version of Figure 4, as suggested.

Reviewer Comment:

Did you attempt to measure free hemoglobin? In addition, did plasma haptoglobin levels change?

Author Response:

We thank the reviewer for this comment. The amount of biological material available (blood) was limited, which restricted the performance of additional analyses, including the measurement of free hemoglobin.

Regarding plasma haptoglobin, attempts were made to quantify it; however, the values obtained did not reach the minimum detection limit of the equipment used. Considering the context of sickle cell anemia, in which there is marked consumption of haptoglobin due to its binding to free hemoglobin, a detectable level of this parameter was not expected.

Therefore, it was not possible to obtain reliable measurements for these markers in the present study.

________________________________________

Closing Remarks

We would like to thank the Editor and the reviewers once again for their careful evaluation of the manuscript and for the valuable suggestions provided. All comments were carefully considered, and we believe that the revisions made have significantly improved the clarity, consistency, and scientific quality of the work.

We hope that the revised version meets the expectations, and we remain available for any further clarifications that may be required.

Attachment Submitted filename: Response to reviewers.docx

# Editor report: Decision Letter 1

**DOI:** 10.1371/journal.pone.0357180.r003

**Authors:** Fábio Silva

5 Jun 2026

Dear Dr. Corat,

Thank you for submitting your manuscript to PLOS ONE. After careful consideration, we feel that it has merit but does not fully meet PLOS ONE’s publication criteria as it currently stands. Therefore, we invite you to submit a revised version of the manuscript that addresses the points raised during the review process.

Please submit your revised manuscript by Jul 20 2026 11:59PM. If you will need more time than this to complete your revisions, please reply to this message or contact the journal office at plosone@plos.org. When you're ready to submit your revision, log on to https://www.editorialmanager.com/pone/ and select the 'Submissions Needing Revision' folder to locate your manuscript file.

- A letter that responds to each point raised by the academic editor and reviewer(s). You should upload this letter as a separate file labeled 'Response to Reviewers'.
- A marked-up copy of your manuscript that highlights changes made to the original version. You should upload this as a separate file labeled 'Revised Manuscript with Track Changes'.
- An unmarked version of your revised paper without tracked changes. You should upload this as a separate file labeled 'Manuscript'.

If you would like to make changes to your financial disclosure, please include your updated statement in your cover letter. Guidelines for resubmitting your figure files are available below the reviewer comments at the end of this letter.

If applicable, we recommend that you deposit your laboratory protocols in protocols.io to enhance the reproducibility of your results. Protocols.io assigns your protocol its own identifier (DOI) so that it can be cited independently in the future. For instructions see: https://journals.plos.org/plosone/s/submission-guidelines#loc-laboratory-protocols. Additionally, PLOS ONE offers an option for publishing peer-reviewed Lab Protocol articles, which describe protocols hosted on protocols.io. Read more information on sharing protocols at https://plos.org/protocols?utm_medium=editorial-email&utm_source=authorletters&utm_campaign=protocols.

As the corresponding author, your ORCID iD is verified in the submission system and will appear in the published article. PLOS supports the use of ORCID, and we encourage all coauthors to register for an ORCID iD and use it as well. Please encourage your coauthors to verify their ORCID iD within the submission system before final acceptance, as unverified ORCID iDs will not appear in the published article. *Only*  the individual author can complete the verification step; PLOS staff *cannot*  verify ORCID iDs on behalf of authors.

We look forward to receiving your revised manuscript.

Kind regards,

Fábio Henrique Silva, Ph.D.

Academic Editor

PLOS One

Journal Requirements:

If the reviewer comments include a recommendation to cite specific previously published works, please review and evaluate these publications to determine whether they are relevant and should be cited. There is no requirement to cite these works unless the editor has indicated otherwise.

Please review your reference list to ensure that it is complete and correct. If you have cited papers that have been retracted, please include the rationale for doing so in the manuscript text, or remove these references and replace them with relevant current references. Any changes to the reference list should be mentioned in the rebuttal letter that accompanies your revised manuscript. If you need to cite a retracted article, indicate the article’s retracted status in the References list and also include a citation and full reference for the retraction notice.

Additional Editor Comments :

Dear Authors,

Thank you for submitting the revised version of the manuscript. The revision has improved the clarity of the study, particularly regarding the animal model, HPLC analysis, experimental groups, and rationale for using haptoglobin 1. However, a few points still need to be addressed before the manuscript can be considered for acceptance.

1. Revise graphical presentation of quantitative data

The previous reviewer suggested that graphs should display individual data points rather than only closed bars. In the current version, Figure 4 still appears to be presented as bar graphs with mean ± SD. Please revise Figure 4 to show individual data points for each animal, with mean ± SD or median and dispersion as appropriate. Given the final n = 3 per group, showing individual values is essential for transparent interpretation.

2. Add a final limitations paragraph

Please add a dedicated limitations paragraph at the end of the Discussion. This paragraph should explicitly address the main limitations of the study, including the small final sample size, the absence of direct quantification of circulating free hemoglobin and free heme, the lack of reliable plasma haptoglobin measurement, and the limited inflammatory assessment. In particular, the manuscript currently discusses possible anti-inflammatory effects. However, total WBC counts did not differ significantly between groups, differential leukocyte counts were not performed, and neutrophils, cytokines, or tissue inflammatory markers were not quantified. Therefore, conclusions regarding inflammation should be interpreted cautiously and primarily based on histopathological observations.

3. Contextualize the findings with previous haptoglobin-based studies in SCD models

Please briefly discuss previous evidence from sustained haptoglobin treatment in sickle cell mice, particularly the study “Sustained treatment of sickle cell mice with haptoglobin increases HO-1 and H-ferritin expression and decreases iron deposition in the kidney without improvement in kidney function” published in British Journal of Haematology in 2016.

This study is relevant because haptoglobin treatment increased markers related to heme and iron handling, including HO-1 and H-ferritin, and reduced renal iron deposition, but did not improve hematological parameters or kidney function. Since the present manuscript reports improvement in hematological parameters after haptoglobin overexpression, the authors should briefly discuss how their findings compare with this previous study.

The discussion should address whether differences between the studies may be related to the therapeutic strategy used, gene-based haptoglobin overexpression versus sustained haptoglobin protein treatment, treatment duration, disease model, timing of analysis, or sample size. This comparison will help readers interpret whether the hematological improvement observed in the present study reflects a distinct effect of the experimental approach or should be considered exploratory and requiring further confirmation.

[Note: HTML markup is below. Please do not edit.]

[NOTE: If reviewer comments were submitted as an attachment file, they will be attached to this email and accessible via the submission site. Please log into your account, locate the manuscript record, and check for the action link "View Attachments". If this link does not appear, there are no attachment files.]

To ensure your figures meet our technical requirements, please review our figure guidelines: https://journals.plos.org/plosone/s/figures

You may also use PLOS’s free figure tool, NAAS, to help you prepare publication quality figures: https://journals.plos.org/plosone/s/figures#loc-tools-for-figure-preparation.

NAAS will assess whether your figures meet our technical requirements by comparing each figure against our figure specifications.

# Author comment: Author response to Decision Letter 2

**DOI:** 10.1371/journal.pone.0357180.r004

19 Jul 2026

We would like to thank the reviewers for their careful evaluation of our manuscript, as well as for their valuable comments and suggestions. We sincerely appreciate the time and effort dedicated to the assessment of our work.

All comments were carefully considered, and the manuscript has been revised accordingly. The changes made are highlighted in the revised version, and detailed responses to each point raised are provided below.

We believe that these revisions have significantly improved the clarity and overall quality of the manuscript.

________________________________________

Editor’s Comment:

Please include the following items when submitting your revised manuscript: a response letter, a marked-up version of the manuscript, and a clean version of the manuscript.

Authors’ Response:

All requested documents have been properly prepared and submitted according to the instructions: (i) the response letter to the reviewers, (ii) the revised manuscript with tracked changes, and (iii) the final clean version of the manuscript without markings.

Editor’s Comment:

If you wish to make changes to your financial disclosure, please include the updated version in your cover letter. We also recommend depositing your laboratory protocols in protocols.io.

Authors’ Response:

The cover letter has been reviewed and updated as necessary. Regarding the recommendation to make protocols/data available, we inform that these have been deposited in the institutional repository of the University of Campinas (REDU), with the corresponding identifier: https://doi.org/10.25824/redu/KYLRCN

Editor’s Comment:

Please ensure that your manuscript meets the PLOS ONE style requirements, including file naming conventions.

Authors’ Response:

The manuscript has been reviewed and adjusted to comply with PLOS ONE style requirements, including formatting and file naming, as instructed.

Editor’s Comment:

Provide additional information in the Methods section regarding animal experiments, including euthanasia methods, anesthesia/analgesia, and efforts to alleviate suffering.

Author Response:

We thank the reviewer for this comment. The requested information has been included and detailed in the Methods section of the revised manuscript.

Briefly, animals were anesthetized with a combination of ketamine (100 mg/kg) and xylazine (10 mg/kg), ensuring adequate sedation, analgesia, and muscle relaxation for the experimental procedures. For euthanasia, a high anesthetic dose (300 mg/kg ketamine and 30 mg/kg xylazine) was administered, followed by cervical dislocation, in accordance with the guidelines of the Animal Ethics Committee (CEUA) and CONCEA.

In addition, measures were implemented throughout the entire experiment to minimize animal suffering, including continuous monitoring, appropriate anesthesia and analgesia protocols, and procedures conducted by a trained team, in compliance with the 3Rs principles.

Editor’s Comment:

A revised Funding Statement is requested, including all sources of support, as well as the inclusion of the sentence “No additional external funding was received for this study.”

Author Response:

We thank the reviewer for this comment. The Funding Statement has been revised and updated to include all sources of support received during the conduct of the study, in accordance with the journal guidelines. The requested sentence (“No additional external funding was received for this study”) has also been included. The updated Funding Statement has been incorporated into the cover letter.

Editor’s Comment:

Please indicate the role of the funders in the study, including the appropriate statement in the cover letter.

Author Response:

We thank the reviewer for this comment. The statement regarding the role of the funders has been included in the cover letter, as requested. Specifically, we state that the funders had no role in study design, data collection and analysis, decision to publish, or preparation of the manuscript.

Editor’s Comment:

Please remove funding information from the Acknowledgements section and update the Funding Statement.

Author Response:

We thank the reviewer for this comment. The funding-related information has been removed from the Acknowledgements section of the manuscript, as instructed.

The Funding Statement has been revised and updated in accordance with the journal guidelines, and the corrected version has been included in the cover letter for the corresponding updates in the submission system.

Editor’s Comment:

Please confirm and, if necessary, update the Data Availability Statement, ensuring access to the minimum dataset required to replicate the study.

Author Response:

We thank the reviewer for this comment. The Data Availability Statement has been revised to ensure compliance with the PLOS ONE guidelines.

The minimum dataset required for replication of the study results has been made publicly available in an institutional repository (REDU – University of Campinas), with the corresponding link/DOI: https://doi.org/10.25824/redu/KYLRCN

In addition, we confirm that all data relevant to the interpretation of the results are included within the manuscript, the supplementary files, and/or the indicated repository.

Editor’s Comment:

Figure 3 contains copyrighted images. Please obtain permission or replace the figure with content compliant with the CC BY 4.0 license.

Author Response:

We thank the reviewer for this comment. Figure 3 has been replaced with a new original version, without the use of any previously copyrighted material, and is fully compliant with the CC BY 4.0 license requirements.

Editor’s Comment:

Revise graphical presentation of quantitative data. The previous reviewer suggested that graphs should display individual data points rather than only closed bars. In the current version, Figure 4 still appears to be presented as bar graphs with mean ± SD. Please revise Figure 4 to show individual data points for each animal, with mean ± SD or median and dispersion as appropriate. Given the final n = 3 per group, showing individual values is essential for transparent interpretation.

Author Response:

We thank the editor for this comment. Figure 4 has been replaced with a new original version as the reviewer suggestion.

Editor’s Comment:

Please add a dedicated limitations paragraph at the end of the Discussion. This paragraph should explicitly address the main limitations of the study, including the small final sample size, the absence of direct quantification of circulating free hemoglobin and free heme, the lack of reliable plasma haptoglobin measurement, and the limited inflammatory assessment. In particular, the manuscript currently discusses possible anti-inflammatory effects. However, total WBC counts did not differ significantly between groups, differential leukocyte counts were not performed, and neutrophils, cytokines, or tissue inflammatory markers were not quantified. Therefore, conclusions regarding inflammation should be interpreted cautiously and primarily based on histopathological observations.

Author Response:

We thank the editor for this comment. The limitations paragraphs have been added.

Lines 568-602. In resume: the interpretation of the present findings should consider the inherent limitations of severe sickle cell disease (SCD) animal models, including their low reproductive performance, high susceptibility to experimental manipulation, reduced survival, and the biological variability associated with bone marrow transplantation-based models. To minimize this variability, only animals with comparable sickle hemoglobin (HbS) levels were included, improving the internal consistency of the study despite the limited sample size.

An additional limitation was the small volume of blood that could be collected from severely anemic animals, restricting complementary analyses such as inflammatory profiling, longitudinal assessment of circulating cell-free hemoglobin, and pharmacokinetic evaluation of haptoglobin (Hp). Furthermore, circulating Hp concentrations could not be reliably quantified, likely due to the rapid consumption of Hp following complex formation with free hemoglobin during chronic intravascular hemolysis, resulting in concentrations below the detection limit of the analytical method. Therefore, future studies employing more sensitive analytical techniques will be necessary to characterize Hp pharmacokinetics and further elucidate the relationship between circulating Hp levels, free Hb scavenging, and therapeutic efficacy.

Editor’s Comment:

Contextualize the findings with previous haptoglobin-based studies in SCD models

Please briefly discuss previous evidence from sustained haptoglobin treatment in sickle cell mice, particularly the study “Sustained treatment of sickle cell mice with haptoglobin increases HO-1 and H-ferritin expression and decreases iron deposition in the kidney without improvement in kidney function” published in British Journal of Haematology in 2016.

This study is relevant because haptoglobin treatment increased markers related to heme and iron handling, including HO-1 and H-ferritin, and reduced renal iron deposition, but did not improve hematological parameters or kidney function. Since the present manuscript reports improvement in hematological parameters after haptoglobin overexpression, the authors should briefly discuss how their findings compare with this previous study.

The discussion should address whether differences between the studies may be related to the therapeutic strategy used, gene-based haptoglobin overexpression versus sustained haptoglobin protein treatment, treatment duration, disease model, timing of analysis, or sample size. This comparison will help readers interpret whether the hematological improvement observed in the present study reflects a distinct effect of the experimental approach or should be considered exploratory and requiring further confirmation.

Author Response:

We thank the editor for this comment. Paragraphs contextualizing previous studies have been added.

Lines 526-567. In General, a previous study evaluating repeated administration of synthetic haptoglobin (Hp) in Townes sickle cell mice reported modest therapeutic effects, including increased HO-1 and ferritin-H expression, reduced renal iron deposition, and a trend toward decreased hepatic infarction (32). In contrast, our gene therapy approach induced sustained Hp expression and produced broader therapeutic benefits, including significant improvement in splenic histology and erythroid hematological parameters.

These differences are likely attributable to the distinct experimental models and treatment strategies. While continuous Hp expression attenuated the progression of hemolysis-associated tissue damage, it did not completely prevent disease progression, indicating that Hp supplementation alone is insufficient to fully restore homeostasis.

Given the continuous release of free hemoglobin during chronic hemolysis, combining sustained Hp expression through gene therapy with the initial administration of exogenous recombinant Hp may represent a more effective therapeutic strategy by providing both immediate and long-term extracellular hemoglobin scavenging.

Reviewer Comment:

What is the number of mice used? [...] In addition, the graphs could be presented as individual points instead of bar graphs.

Author Response:

We thank the reviewer for this comment. Information regarding the number of animals has been included and detailed in the revised manuscript.

Six animals were transplanted in each experimental group. In both groups, deaths occurred, with one death recorded in the treated group and two in the control group. In the treated group, one animal showed a high level of chimerism (~95%), while another presented a very low level and was therefore excluded from the analysis. Thus, three animals with intermediate levels of chimerism (mean approximately 75%) were included in the analysis.

In the control group, animals with chimerism levels comparable to those observed in the treated group were selected, totaling three animals for analysis.

Additionally, the number of animals (n = 3) has been included in the figure legends. We consider that this information allows appropriate interpretation of the presented results.

Reviewer Comment:

Why was haptoglobin 1 overexpression chosen instead of haptoglobin 2? Please justify this choice and discuss the clinical implications in the context of Hp1 versus Hp2 comparison.

Author Response:

We thank the reviewer for this comment. The choice of haptoglobin phenotype Hp1 was based on literature evidence demonstrating the greater efficiency of this phenotype in neutralizing the deleterious effects of free hemoglobin.

Studies indicate that Hp1-1 exhibits higher antioxidant capacity compared to Hp2-2, promoting the formation of more stable Hb-Hp complexes with more efficient clearance via endocytosis. In contrast, complexes formed with Hp2-2 are associated with increased generation of reactive oxygen species, thereby enhancing the potential for oxidative damage to lipids, proteins, and DNA.

Therefore, considering the context of chronic hemolysis observed in sickle cell anemia, Hp1 was considered more appropriate as a therapeutic strategy due to its greater efficiency in reducing oxidative stress and its potential tissue-protective effects (LEVY et al., 2010; PIRES; BIERHALZ; MORAES, 2015; PIRES; BERTHIAUME; PALMER, 2023; QUIMBY; HAMBLETON; LANDIS, 2015).

The clinical implications of this choice have been included and further discussed in the revised version of the manuscript.

Reviewer Comment:

Describe how and when the SS bone marrow graft was confirmed, which method was used, and discuss the impact of circulating wild-type red blood cells at the time of treatment.

Author Response:

We thank the reviewer for this comment. The engraftment was confirmed by high-performance liquid chromatography (HPLC) using the Variant II automated system (Bio-Rad). Peripheral blood samples were collected via submandibular puncture (cheek pouch), subsequently aspirated, injected into the analytical system, and applied to a cation-exchange column. A programmed buffer gradient enabled the separation of different hemoglobin fractions based on their ionic interactions with the column. Eluted fractions were detected by photometry at 415 nm, identified by their characteristic retention times, and quantified by peak area integration.

The transplanted models were considered to exhibit a severe sickle cell anemia phenotype 60 days post-transplantation, with an HbS proportion above 70%, as described by Iannone et al. (2001).

Regarding the presence of wild-type red blood cells, it is important to note that gamma-irradiated erythrocytes exhibit a significantly reduced lifespan, estimated at approximately 10 days, as reported in the literature. Therefore, at 60 days post-transplantation, animals predominantly present erythrocytes derived from the transplanted bone marrow, expressing physiological and molecular features consistent with sickle cell anemia. Additionally, in the absence of transplantation, animals do not survive beyond 7–10 days due to the lack of functional erythrocytes.

These points have been included and discussed in the revised manuscript.

Reviewer Comment:

Indicate whether inflammatory parameters (e.g., leukocytes, neutrophils) were assessed after treatment and discuss their relevance to the therapeutic potential of haptoglobin.

Author Response:

We thank the reviewer for this comment. Differential leukocyte counts were not performed in the present study. Regarding total leukocyte counts, high variability among samples was observed, and no statistically significant differences were identified between groups.

On the other hand, a significant increase in platelet counts was observed in the treated animals.

We emphasize that a more precise evaluation of the inflammatory profile would require the assessment of specific markers, such as cytokines, which were not performed in this study; however, this approach will be considered in future investigations.

Nevertheless, as described in the literature (Hurtado et al., 2023), hemolytic conditions are associated with increased levels of free heme, which can promote oxidative stre

Attachment Submitted filename: Response_to_reviewers_auresp_2.docx

# Editor report: Decision Letter 2

**DOI:** 10.1371/journal.pone.0357180.r005

**Authors:** Fábio Silva

13 Aug 2026

Therapeutic potential of haptoglobin in a murine model of sickle cell anemia

PONE-D-25-51689R2

Dear Dr. Corat,

We’re pleased to inform you that your manuscript has been judged scientifically suitable for publication and will be formally accepted for publication once it meets all outstanding technical requirements.

Within one week, you’ll receive an e-mail detailing the required amendments. When these have been addressed, you’ll receive a formal acceptance letter and your manuscript will be scheduled for publication.

An invoice will be generated when your article is formally accepted. Please note, if your institution has a publishing partnership with PLOS and your article meets the relevant criteria, all or part of your publication costs will be covered. Please make sure your user information is up-to-date by logging into Editorial Manager at Editorial Manager® and clicking the ‘Update My Information' link at the top of the page. For questions related to billing, please contact billing support.

If your institution or institutions have a press office, please notify them about your upcoming paper to help maximize its impact. If they’ll be preparing press materials, please inform our press team as soon as possible -- no later than 48 hours after receiving the formal acceptance. Your manuscript will remain under strict press embargo until 2 pm Eastern Time on the date of publication. For more information, please contact onepress@plos.org.

Kind regards,

Fábio Henrique Silva, Ph.D.

Academic Editor

PLOS One

Additional Editor Comments (optional):

Reviewers' comments:

# Editor report: Acceptance letter

**DOI:** 10.1371/journal.pone.0357180.r006

**Authors:** Fábio Silva

PONE-D-25-51689R2

PLOS One

Dear Dr. Corat,

I'm pleased to inform you that your manuscript has been deemed suitable for publication in PLOS One. Congratulations! Your manuscript is now being handed over to our production team.

At this stage, our production department will prepare your paper for publication. This includes ensuring the following:

* All references, tables, and figures are properly cited

* All relevant supporting information is included in the manuscript submission,

* There are no issues that prevent the paper from being properly typeset

You will receive further instructions from the production team, including instructions on how to review your proof when it is ready. Please keep in mind that we are working through a large volume of accepted articles, so please give us a few days to review your paper and let you know the next and final steps.

Lastly, if your institution or institutions have a press office, please let them know about your upcoming paper now to help maximize its impact. If they'll be preparing press materials, please inform our press team within the next 48 hours. Your manuscript will remain under strict press embargo until 2 pm Eastern Time on the date of publication. For more information, please contact onepress@plos.org.

You will receive an invoice from PLOS for your publication fee after your manuscript has reached the completed accept phase. If you receive an email requesting payment before acceptance or for any other service, this may be a phishing scheme. Learn how to identify phishing emails and protect your accounts at https://explore.plos.org/phishing.

If we can help with anything else, please email us at customercare@plos.org.

Thank you for submitting your work to PLOS One and supporting open access.

Kind regards,

PLOS ONE Editorial Office Staff

on behalf of

Dr. Fábio Henrique Silva

Academic Editor

PLOS One
